# Liver fraction of circulating alkaline phosphatase is elevated in chronic kidney disease and associates with mortality in patients treated with haemodialysis

**DOI:** 10.1093/ckj/sfag078

**Published:** 2026-03-11

**Authors:** Dieter Smout, Mathias Haarhaus, Patrick D’Haese, Hanne Skou Jørgensen, Kathleen Claes, Bert Bammens, Amaryllis H Van Craenenbroeck, Björn Meijers, Viviane Van Hoof, Etienne Cavalier, Pieter Evenepoel

**Affiliations:** Department of Microbiology, Immunology and Transplantation, Nephrology and Renal Transplantation Research Group, Katholieke Universiteit Leuven, Leuven, Belgium; Department of Nephrology and Renal Transplantation, University Hospitals Leuven, Leuven, Belgium; Division of Renal Medicine, Department of Clinical Science, Intervention and Technology, Karolinska Institutet, Karolinska Universitetssjukhuset, Stockholm, Sweden; Diaverum Sweden AB, Malmö, Sweden; Laboratory of Pathophysiology, Department Biomedical Sciences, University of Antwerp, Antwerp, Belgium; Department of Renal Medicine, Aarhus University Hospital, Aarhus, Denmark; Department of Clinical Medicine, Aarhus University, Aarhus, Denmark; Department of Clinical Medicine, Aalborg University, Aalborg, Denmark; Department of Microbiology, Immunology and Transplantation, Nephrology and Renal Transplantation Research Group, Katholieke Universiteit Leuven, Leuven, Belgium; Department of Nephrology and Renal Transplantation, University Hospitals Leuven, Leuven, Belgium; Department of Microbiology, Immunology and Transplantation, Nephrology and Renal Transplantation Research Group, Katholieke Universiteit Leuven, Leuven, Belgium; Department of Nephrology and Renal Transplantation, University Hospitals Leuven, Leuven, Belgium; Department of Microbiology, Immunology and Transplantation, Nephrology and Renal Transplantation Research Group, Katholieke Universiteit Leuven, Leuven, Belgium; Department of Nephrology and Renal Transplantation, University Hospitals Leuven, Leuven, Belgium; Department of Microbiology, Immunology and Transplantation, Nephrology and Renal Transplantation Research Group, Katholieke Universiteit Leuven, Leuven, Belgium; Department of Nephrology and Renal Transplantation, University Hospitals Leuven, Leuven, Belgium; Faculty of Medicine and Health Sciences, University of Antwerp, Antwerp, Belgium; Department of Clinical Chemistry, CIRM, University of Liège, CHU de Liège, Belgium; Department of Microbiology, Immunology and Transplantation, Nephrology and Renal Transplantation Research Group, Katholieke Universiteit Leuven, Leuven, Belgium; Department of Nephrology and Renal Transplantation, University Hospitals Leuven, Leuven, Belgium

**Keywords:** alkaline phosphatase, CKD, CKD-MBD, inflammation, parathyroid hormone

## Abstract

**Background:**

Serum total alkaline phosphatase (ALP) is a robust predictor of all-cause mortality in both the general population and in patients with chronic kidney disease (CKD). Individual ALP isozymes and isoforms exert specific physiological functions and may therefore inform separately on different disease processes and risks related to these. Serum ALP consists predominantly of bone and liver isoforms, with a smaller contribution from the intestinal isoenzyme. We aimed to characterize the impact of CKD on serum ALP fractions and to identify clinical and biochemical correlates.

**Methods:**

Serum ALP liver, bone and intestinal fractions were analysed by electrophoresis in 523 individuals across stages of CKD (stage G1–2: *n* = 90; G3: *n* = 100; G4–5: *n* = 139; G5D: *n* = 194) and in 21 kidney-healthy controls. Associations with demographics and parameters of mineral metabolism and inflammation were examined. In haemodialysis patients, we further explored the association between ALP isozymes and all-cause mortality.

**Results:**

Total ALP levels increased significantly across stages of CKD, with liver, bone and intestinal fractions all contributing. In patients with CKD G5D, circulating levels of all three fractions were more than two-fold higher than in controls. Bone ALP associated with PTH while liver ALP associated with C-reactive protein, both independent of demographics and kidney function. Higher liver ALP levels associated with increased mortality risk, independent of traditional risk factors and inflammation.

**Conclusions:**

In patients with CKD, elevations in liver, bone and intestinal ALP fractions reflect distinct biological pathways and may differentially inform on risk related to different disease processes. While bone ALP reflects bone metabolism, liver ALP is linked with inflammation. The elevation in CKD and the independent association of liver ALP fraction with mortality in patients treated with haemodialysis calls for additional clinical and mechanistic studies.

## GRAPHICAL ABSTRACT

**GRAPHICAL ABSTRACT:**
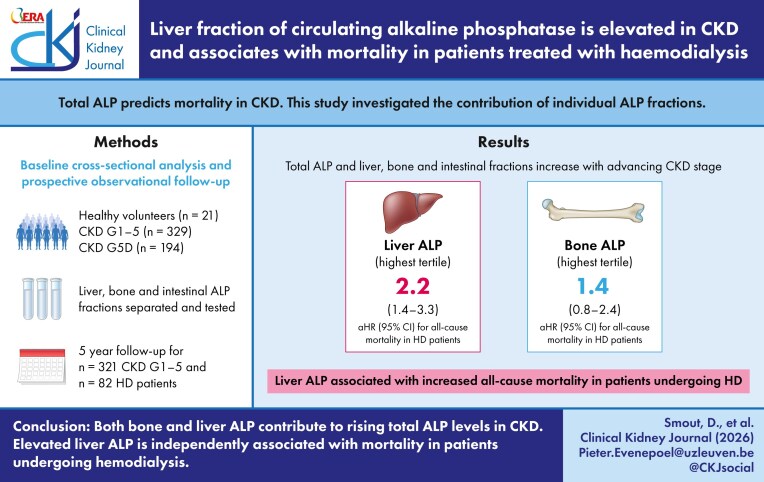

KEY LEARNING POINTS **What was known:**

- Total alkaline phosphatase (ALP) is a robust predictor of mortality in chronic kidney disease (CKD) but the mechanisms underlying this association remain poorly defined.
- Circulating ALP consists mainly of bone and liver isoforms, with smaller contributions from intestinal ALP.
- Published studies of ALP fractions in CKD yield inconsistent findings and are limited by small sample sizes.

**This study adds:**

- Total ALP increases progressively across CKD stages, driven by increases in liver, bone and intestinal fractions.
- While bone ALP associates with mineral metabolism, liver ALP is closely linked to inflammation.
- Elevated liver ALP independently associated with 5-year mortality risk in patients treated with haemodialysis.

**Potential impact:**

- Clinicians should recognize that elevated total ALP in CKD may reflect not only increased bone turnover but also inflammation.
- Further studies should elucidate the mechanisms driving liver ALP and assess its potential as a therapeutic or prognostic target in patients with CKD.
- Fraction-specific assays for ALP beyond bone ALP may prove useful in clinical practice.

## INTRODUCTION

Chronic kidney disease (CKD) is a global health concern, affecting >10% of the world’s population [1]. Despite major diagnostic and therapeutic advances in recent years, patients with CKD continue to face disproportionately high morbidity and mortality, largely driven by cardiovascular disease and infections [2, 3]. Traditional risk factors only partly account for this excess risk. Elevated circulating alkaline phosphatase (ALP) activity has consistently been associated with adverse outcomes in both the general population [4, 5] and in patients with CKD [6–14], sparking interest in ALP as both a biomarker and a potential therapeutic target. However, the underlying mechanisms remain incompletely understood.

ALP consists of four genetically distinct isoenzymes: tissue-non-specific (TNALP), intestinal, placental and germ cell ALP [8]. TNALP is expressed in different organs, including liver, bone, kidney, brain, blood vessels, intestines and leucocytes. Although TNALP is encoded by a single gene, post-translational modifications, mainly glycosylation, vary across tissues, generating multiple isoforms. In serum, the most prominent TNALP isoforms derive from bone and the liver, together accounting for >90% of total ALP activity [15]. The remaining ALP, ranging from 1% to 10%, is primarily of intestinal ALP origin [16, 17].

Various techniques and methods are used for the separation and quantification of ALP isozymes and isoforms, each having inherent limitations [18]. Electrophoretic assays can be used for visualizing and investigating the origin of increased serum total ALP activities by separating and quantifying liver, bone, intestinal and other ALP fractions [19, 20]. Bone ALP can be directly measured with immunoassays and has been extensively investigated with respect to its structural, kinetic, immunologic, functional and clinical properties using high-performance liquid chromatography (HPLC) [16, 21–23]. Liver ALP is less well characterized, and while elevations in the circulating liver ALP fraction are assumed to originate from the hepatobiliary system, other tissues, including the kidneys, brain and leucocytes, may also contribute to this fraction [15, 17, 24].

The key role of bone ALP in bone mineralization is well established and it has also been implicated in pathological calcification of soft tissues [7, 25]. In contrast, there is emerging evidence linking liver and intestinal ALP with inflammation [7, 8]. These observations are particularly relevant in CKD, where chronic inflammation is highly common and associated with poor outcomes [26].

So far, studies on ALP fractions in patients with CKD have yielded inconsistent findings, were limited by rather small sample sizes and did not cover the whole spectrum of CKD severity [27–31]. This study examined serum activity levels of ALP fractions in a sizeable cohort of patients across CKD stages. As a secondary aim, we explored clinical and biochemical determinants of these fractions and their association with mortality.

## MATERIALS AND METHODS

### Study population and design

This study comprises a cross-sectional analysis and a prospective observational substudy. We compiled data from several completed and ongoing clinical cohort studies at the University Hospitals Leuven in Belgium, designed to identify novel cardiovascular risk factors in adults with CKD G1–5D [32, 33]. These cohorts included patients with CKD G1–5 (S63818, S51935), patients on haemodialysis (HD; S33350) and kidney transplant candidates (S52091) without specific exclusion criteria.

For the present analysis, adults enrolled between February 2006 and June 2012 with available data on ALP isozymes were eligible (*n* = 577). Patients with a documented history of liver disease or malnutrition, based on International Classification of Diseases, Ninth Revision diagnostic codes, were excluded (*n* = 33). Demographics, comorbidities and medication use were extracted from electronic health records.

All studies were performed according to the Declaration of Helsinki and approved by the ethics committee of the Universitair Ziekenhuis/Katholieke Universiteit Leuven (S63818, S51935, S33350, S52091). Informed consent was obtained from all participants.

### Biochemical measurements

In patients with CKD not yet on dialysis and in patients receiving peritoneal dialysis (PD), non-fasted, morning blood samples were collected at baseline. In patients receiving HD, baseline samples were collected prior to the mid-week dialysis session. After centrifugation, serum was aliquoted and stored at −80°C until analysis.

Total ALP activity was measured by kinetic assay (Dimension Vista, Siemens, Munich, Germany). Reference upper limits of normal were 128 U/l (men <60 years of age), 98 U/l (women <60 years of age), 119 U/l (men ≥60 years of age) and 141 U/l (women ≥60 years of age). Intra- and interassay variability was <5%.

ALP fractions were separated by agarose gel electrophoresis (ISOPAL, Beckman Europe, Analis S.A., Namur, Belgium), as previously described [19, 20]. Briefly, ALP fractions were quantified indirectly by densitometric scanning of untreated samples, samples treated with neuraminidase and polyclonal anti-intestinal ALP antiserum, and samples heated at 65°C for 10 minutes. Additional measurements after treatment with monoclonal anti-placental ALP antiserum, phospholipase C or monospecific anti-liver/bone/kidney ALP antiserum were performed when source identification remained uncertain.

Five fractions were distinguished: liver, bone, intestinal, high molecular weight and intestinal variant. For analysis, the high molecular weight was grouped with the liver fraction and intestinal variant with intestinal ALP. Intra-assay coefficients of variance (CVs) were 2% at 90 U/l for liver ALP, 2% at 37 U/l for bone ALP and 24% at 3 U/l for intestinal ALP; interassay CVs were 3% at 60 U/l, 7% at 29 U/l and 42% at 5 U/l, respectively. ALP fractions were expressed as U/l by multiplying the relative fraction (%) by total ALP activity.

Serum 1-84 parathyroid hormone (PTH) was measured by an in-house immunoradiometric assay (reference interval: 3–40 pg/ml) [34]. Other biochemical parameters—including creatinine, haemoglobin, calcium, phosphate, albumin, C-reactive protein (CRP), aspartate aminotransferase (AST), alanine aminotransferase (ALT), gamma-glutamyl transferase (GGT), ferritin and cholesterol—were measured using the Modular P system (Roche, Basel, Switzerland). All assays reported intra- and interassay variation <15%. The estimated glomerular filtration rate (eGFR) was calculated with the 2009 Chronic Kidney Disease Epidemiology Collaboration (CKD-EPI) equation. Patients not on dialysis were categorized as CKD G1–2, G3 or G4–5 using cut-offs of >60, 30–60 and <30 ml/min/1.73 m^2^, respectively.

### Prospective data

Data on overall mortality and cause of death up to 5 years after the index date were extracted from electronic medical records. Causes of death were classified as cardiovascular, infection-related or other. If documentation was unavailable or cause was listed as unknown, the event was categorized as unknown. Patients recruited as kidney transplant candidates were excluded from longitudinal analyses.

### Statistical analysis

Continuous variables are presented as mean [standard deviation (SD)] or median [interquartile range (IQR)], depending on distribution. Group comparisons used analysis of variance (ANOVA), Kruskal–Wallis or chi-squared test, as appropriate. Locally estimated scatter plot smoothing (LOESS) of total ALP and its fractions versus eGFR were fitted with a bandwidth of 0.8. Associations between ALP isoenzymes and clinical variables were assessed with Spearman correlation coefficients. Skewed parameters were log-transformed before parametric analyses.

To investigate potential determinants of ALP fractions, we used univariable and multivariable linear regression in an aetiological framework with covariates selected a priori based on biological plausibility and previous literature [35]. These included age, sex, body mass index (BMI) and biochemical parameters of CKD–mineral and bone disorder (CKD-MBD) {calcium, phosphate, PTH, bicarbonate, 25-hydroxyvitamin D [25(OH)D]} and inflammation (CRP). Multivariable analyses were conducted on complete case data (*n* = 260 for CKD G1–5 and *n* = 146 for CKD G5D). Due to the absence of intestinal ALP expression and high variability in intestinal ALP, we reported exploratory univariate analyses with intestinal ALP as logistic regressions (present or absent) and omitted intestinal ALP from multivariate analyses.

Survival analysis for all-cause mortality was performed with Kaplan–Meier curves and Cox proportional hazards models. Patients were censored at 5 years, kidney transplantation or loss to follow-up. A two-sided *P*-value <.05 was considered statistically significant. All analyses were conducted in R version 4.4.1 (R Foundation for Statistical Computing, Vienna, Austria).

## RESULTS

### Study population characteristics

The study population included 21 healthy adults and 523 adults with CKD G1–5D. Among the 329 non-dialysis CKD patients, 90 were stage G1–2 (27.4%), 100 stage G3 (30.4%) and 139 stage G4–5 (42.2%; Supplementary Table S1). Of the 194 patients with CKD G5D, 163 (84%) were receiving HD and 31 (16%) PD.

The mean age of patients was 62 years and 58% were male. Healthy controls and patients with CKD G1–2 were significantly younger than patients with more advanced CKD. The mean BMI was 25.3 kg/m^2^. Diabetes mellitus was present in 24% and 34% had a history of cardiovascular disease. Among patients receiving dialysis, the median dialysis vintage was 28 months (IQR 17–41).

### Circulating ALP fractions across CKD stages

**Figure 1:**
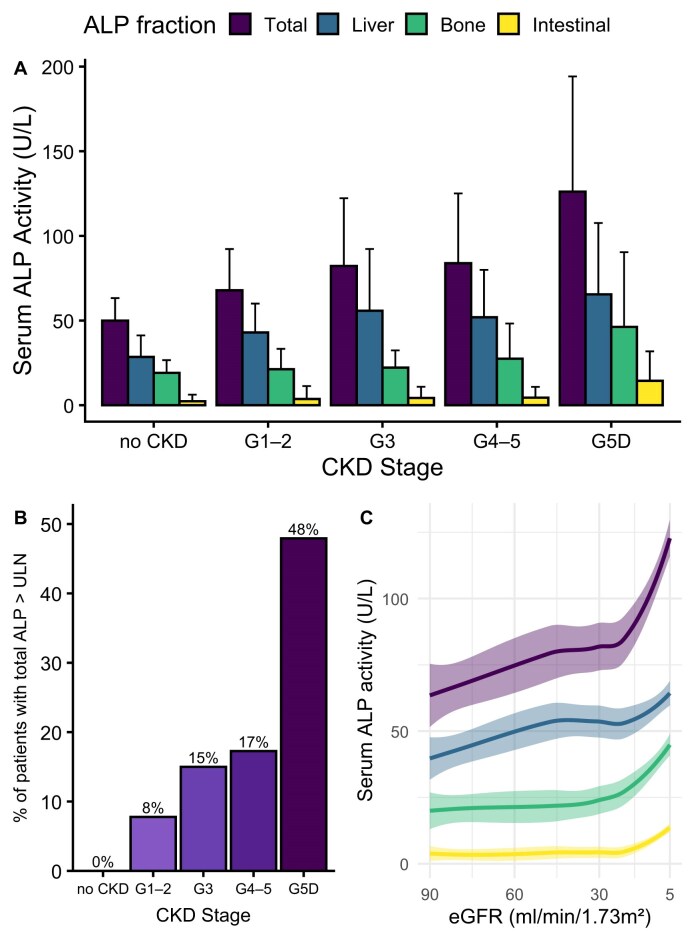
Serum ALP fractions increase with declining kidney function. **(A)** ALP fractions across stages of CKD. Bars show mean ± SD. **(B)** Percentage of patients with total ALP above the upper limit of normal (ULN) of the age- and sex-specific reference range. **(C)** LOESS regression of ALP isozyme fractions and eGFR (CKD-EPI 2009) with 95% CI.

Serum total ALP as well as levels of the major fractions (liver, bone and intestinal) increased with advancing CKD stage (ANOVA *P*-value <.001 for all; Fig. 1A). None of the kidney-healthy adults showed elevated total ALP, but this was observed with increasing frequency across CKD stages: 8% in patients with CKD G1–2, 15% in CKD G3, 17% in CKD G4–5 and 48% in CKD G5D (Fig. 1B). Total ALP increased progressively with declining eGFR, with a steeper rise observed at lower eGFR levels (Fig. 1C). Liver ALP increased steadily from early-stage CKD, while bone and especially intestinal ALP increased predominantly in advanced CKD, coinciding with the pronounced increase in total ALP at lower eGFRs. In kidney failure, liver ALP was 2.2 times higher compared with healthy controls. Distribution was similar in men and in women.

Intestinal ALP was detectable in 129 of 329 non-dialysis CKD patients (39%) and in 123 of 194 patients on dialysis (63%). Patients treated with PD had significantly higher intestinal ALP levels than those treated with HD (21 versus 8 U/l; *P* = .005).

### Determinants of circulating total ALP and fractions

Patients in the highest tertile of total ALP were characterized by worse kidney function and significantly higher levels of PTH, phosphate and CRP but did not differ in the prevalence of comorbidities such as diabetes, cardiovascular disease, malignancy or autosomal dominant polycystic kidney disease (ADPKD) (Table 1). To account for potential confounding by CKD stage, we stratified patients by tertiles of total ALP within each stage. Across all stages, PTH and CRP were consistently higher in the highest tertile of total ALP (*P* < .001; Supplementary Table S2).

**Table 1:** Patient characteristics across tertiles of total ALP.

	Total ALP tertiles				
Characteristics	Overall (*N* = 544)	Tertile 1 (<67 U/l)	Tertile 2 (67–102 U/l)	Tertile 3 (>102 U/l)	*P*-value
Liver ALP (U/L)	48 (33–66)	31 (24–38)	51 (41–58)	74 (60–97)	**<.001**
Liver ALP (% of total)	61 (50–71)	62 (52–71)	64 (50–73)	58 (45–69)	**.008**
Bone ALP (U/l)	24 (17–37)	16 (12–20)	23 (19–30)	43 (32–58)	**<.001**
Bone ALP (% of total)	31 (24–40)	31 (25–39)	29 (24–37)	33 (23–43)	.064
Intestinal ALP (U/l)	0 (0–12)	0 (0–6)	0 (0–13)	8 (0–21)	**<.001**
Intestinal ALP (% of total)	0 (0–13)	0 (0–11)	0 (0–15)	6 (0–14)	**.048**
Demographics					
Age (years)	62 (49–73)	60 (47–74)	63 (49–74)	63 (50–71)	.7
Sex (male), *n* (%)	318 (58)	100 (55)	112 (62)	106 (59)	.4
BMI (kg/m^2^)	25.3 (22.8–28.9)	24.7 (22.7–29.5)	25.4 (22.8–28.8)	25.6 (23.1–28.7)	.9
Systolic BP (mmHg)	138 (120–150)	132 (120–150)	135 (120–150)	140 (123–155)	.08
Diastolic BP (mmHg)	80 (70–85)	80 (70–85)	80 (70–85)	77 (70–85)	.6
Diabetes mellitus, *n* (%)	125 (24)	41 (25)	34 (19)	50 (28)	.14
Prevalent CVD, *n* (%)	179 (34)	50 (31)	63 (35)	66 (37)	.4
Malignancy, *n* (%)	55 (11)	16 (9.8)	21 (12)	18 (9.9)	.8
ADPKD, *n* (%)	57 (11)	20 (12)	16 (8.9)	21 (12)	.6
CKD stage, *n* (%)					**<.001**
Healthy	21 (3.9)	19 (10)	2 (1.1)	0 (0)	
G1	41 (7.5)	19 (10)	19 (10)	3 (1.7)	
G2	49 (9.0)	29 (16)	16 (8.8)	4 (2.2)	
G3A	38 (7.0)	11 (6.0)	14 (7.7)	13 (7.2)	
G3B	62 (11)	29 (16)	21 (12)	12 (6.6)	
G4	97 (18)	30 (16)	44 (24)	23 (13)	
G5	42 (7.7)	19 (10)	14 (7.7)	9 (5.0)	
HD	163 (30)	25 (14)	43 (24)	95 (52)	
PD	31 (5.7)	1 (0.5)	8 (4.4)	22 (12)	
Biochemistry					
Creatinine (mg/dl)	2.65 (1.40–6.39)	1.70 (1.00–3.26)	2.53 (1.39–5.91)	5.60 (2.28–7.77)	**<.001**
eGFR (ml/min/1.73 m^2^)	22 (8–48)	35 (15–75)	23 (8–49)	9 (6–25)	**<.001**
Urea (mg/dl)	86 (50–123)	69 (36–109)	92 (52–124)	99 (70–129)	**<.001**
Calcium (mg/dl)	9.30 (9.00–9.60)	9.30 (9.00–9.50)	9.30 (8.90–9.60)	9.30 (9.00–9.80)	.10
Phosphate (mg/dl)	3.60 (3.00–4.20)	3.38 (2.89–3.90)	3.60 (3.00–4.10)	3.96 (3.30–5.10)	**<.001**
Bicarbonate (mmol/l)	24.70 (23.00–26.70)	24.85 (23.15–27.25)	24.35 (23.00–26.20)	24.80 (22.80–27.05)	.13
1-84 PTH (pg/ml)	46 (21–112)	26 (14–51)	37 (20–74)	111 (42–233)	**<.001**
25(OH)D (ng/ml)	28 (19–39)	27 (20–37)	27 (17–38)	30 (20–41)	.11
Urea (24-hour urine; g/day)	18 (13–23)	19 (13–24)	19 (13–23)	17 (12–23)	.4
Proteinuria (24-hour; g/day)	0.26 (0.11–0.72)	0.23 (0.10–0.68)	0.31 (0.12–0.92)	0.26 (0.11–0.66)	.3
Albumin (g/l)	44.4 (41.8–46.9)	45.1 (43.1–47.7)	43.8 (41.1–46.5)	44.0 (41.0–46.2)	**<.001**
GGT (U/l)	22 (17–36)	23 (18–32)	20 (15–32)	26 (17–40)	.06
AST (U/l)	21 (17–26)	22 (18–27)	21 (17–26)	21 (16–25)	.14
ALT (U/l)	19 (14–25)	19 (14–25)	18 (14–24)	19 (14–25)	.6
Bilirubin (mg/dl)	0.50 (0.30–0.60)	0.40 (0.30–0.60)	0.40 (0.30–0.60)	0.50 (0.30–0.60)	.8
Haemoglobin (g/dl)	12.60 (11.60–14.00)	12.90 (11.70–14.30)	12.65 (11.75–14.00)	12.40 (11.30–13.60)	**.02**
WBC (10^9^/l)	6.60 (5.29–7.90)	6.50 (5.20–8.00)	6.50 (5.45–7.80)	6.70 (5.10–7.80)	>.9
CRP (mg/l)	3 (1–6)	1 (1–4)	3 (1–6)	4 (2–8)	**<.001**
Cholesterol (mg/dl)	170 (147–193)	174 (152–194)	171 (146–194)	168 (146–190)	.2
HDL (mg/dl)	49 (41–63)	50 (42–64)	48 (40–61)	51 (40–65)	.3
LDL (mg/dl)	87 (66–104)	88 (68–107)	88 (66–106)	82 (59–99)	**.04**
Triglycerides (mg/dl)	132 (90–188)	125 (87–180)	135 (91–190)	134 (94–190)	.6
Free iron (µg/dl)	74 (56–95)	82 (64–105)	73 (55–94)	67 (51–84)	**<.001**
Transferrin (g/l)	2.31 (2.00–2.60)	2.43 (2.05–2.75)	2.35 (2.02–2.63)	2.18 (1.89–2.43)	**<.001**
Transferrin saturation (%)	22 (18–30)	24 (19–31)	22 (18–29)	22 (17–29)	.13
Ferritin (ng/ml)	186 (88–344)	185 (85–294)	153 (78–281)	223 (104–488)	**.003**
Medication, *n* (%)					
Proton pump inhibitor	125 (24)	28 (17)	42 (23)	55 (30)	**.02**
Oral bicarbonate	41 (7.8)	17 (10)	13 (7.3)	11 (6.1)	.3
Calcium supplement	179 (34)	44 (27)	59 (33)	76 (42)	.01
Phosphate binder (calcium-based	32 (6.1)	2 (1.2)	9 (5.0)	21 (12)	**<.001**
Phosphate binder (non-calcium-based)	55 (11)	3 (1.8)	14 (7.8)	38 (21)	**<.001**
Active vitamin D	100 (18)	16 (8.8)	29 (16)	55 (30)	**<.001**
Vitamin D	117 (22)	39 (24)	30 (17)	48 (27)	.07
Bisphosphonate	18 (3.4)	6 (3.7)	7 (3.9)	5 (2.8)	.8
Cinacalcet	17 (3.3)	0 (0)	5 (2.8)	12 (6.6)	**.002**
Oral iron	127 (24)	27 (17)	48 (27)	52 (29)	**.020**
Statin	246 (47)	73 (45)	90 (50)	83 (46)	.6
ACE inhibitor	213 (41)	73 (45)	88 (49)	52 (29)	**<.001**
Angiotensin receptor blocker	96 (18)	39 (24)	31 (17)	26 (14)	.066
Oral corticosteroids	43 (8.2)	15 (9.2)	13 (7.3)	15 (8.3)	.8
Immunosuppressant	34 (6.5)	12 (7.4)	11 (6.1)	11 (6.1)	.9

Values are presented as median (IQR) unless stated otherwise.

Statistical comparisons across tertiles were performed using the Kruskal–Wallis rank-sum test for continuous variables and Pearson’s chi-squared or Fisher’s exact test for categorical variables, as appropriate.

Significant values in bold.

BALP: bone ALP; LALP: liver ALP; IALP: intestinal ALP; BP: blood pressure; CVD: cardiovascular disease.

Spearman correlation analysis (Table 2) showed that total ALP was directly correlated with PTH, phosphate and CRP and inversely with eGFR. Bone ALP correlated most strongly with PTH, while liver ALP was most strongly correlated with CRP. Results of univariable linear regressions for total, liver, bone and intestinal ALP in CKD G1–5 and G5D are detailed in Supplementary Tables S3 and S4.

**Table 2:** Spearman correlation matrix of ALP isozymes and determinants.

Variable	TALP	LALP	BALP	IALP	Age	BMI	eGFR	HCO_3_^−^	PO_4_^3−^	CRP	PTH	25(OH)D
TALP												
LALP	**0.76** ^b^											
BALP	**0.81** ^b^	**0.41** ^b^										
IALP	**0.27** ^b^	−0.04	0.14									
Age	0.02	0.09	−0.08	0.12								
BMI	0	0.08	−0.09	0.01	0.12							
eGFR	**−0.42** ^b^	**−0.2** ^b^	**−0.38** ^b^	**−0.31** ^b^	**−0.26** ^b^	0.03						
Bicarbonate	−0.02	0.01	−0.02	−0.03	−0.08	0.01	**0.2** ^b^					
Phosphate	**0.25** ^b^	0.09	**0.22** ^b^	**0.25** ^b^	0.08	−0.06	**−0.61** ^b^	**−0.16** ^a^				
CRP	**0.28** ^b^	**0.35** ^b^	0.08	0.08	**0.2** ^b^	0.13	**−0.28** ^b^	−0.09	0.15			
PTH	**0.48** ^b^	**0.26** ^b^	**0.51** ^b^	**0.19** ^b^	0.06	−0.07	**−0.67** ^b^	−0.12	**0.44** ^b^	**0.16** ^a^		
25(OH)D	0.06	−0.02	0.08	0.05	−0.1	**−0.16** ^a^	**−0.19** ^b^	0.15	0.04	−0.14	**0.2** ^b^	

Spearman’s correlation coefficients (*r*) are shown. Significance is indicated using Bonferroni-adjusted *P*-values: ^a^*P* < .05 and ^b^*P* < .01.

Significant values in bold.

TALP: total ALP; BALP: bone ALP; LALP: liver ALP; IALP: intestinal ALP.

Total ALP was independently associated with PTH and CRP in both CKD G1–5 and G5D, after adjustment for age, sex and BMI (Table 3). In CKD G1–5, lower 25(OH)D was identified as another independent determinant of higher total ALP.

**Table 3:** Determinants of total ALP in CKD G1–5 and G5D.

	CKD G1-5				CKD G5D			
Variable	Univariable		Multivariable (R^2^ = 0.2077)		Univariable		Multivariable (*R*^2^ = 0.342)	
	β	*P*-value	β	*P*-value	β	*P*-value	β	*P*-value
Intercept			43.555	**<0.001**			37.020	**<0.001**
Age (years)	0.001	0.558	−0.002	0.297	−0.038	0.102	0.001	0.761
Sex (male)	−0.023	0.613	−0.031	0.529	−0.087	0.224	−0.121	0.084
BMI (kg/m^2^)	0.003	0.471	−0.004	0.451	−0.011	0.147	−0.013	0.083
eGFR (ml/min/1.73 m^2^)	−0.002	**0.001**	−0.001	0.172				
Phosphate (mg/dL)	0.081	**0.007**	0.049	0.156	−0.018	0.456	−0.034	0.190
Bicarbonate (mmol/L)	−0.016	**0.035**	0.005	0.602	0.019	0.143	0.003	0.835
ln 1-84 PTH (pg/mL)	0.074	**<0.001**	0.053	**0.037**	0.188	**<0.001**	0.185	**<0.001**
ln 25(OH)D (ng/mL)	−0.121	**0.007**	−0.111	**0.023**	0.239	**0.001**	0.131	0.074
ln CRP (mg/L)	0.102	**<0.001**	0.133	**<0.001**	0.083	**0.027**	0.112	**0.003**

Data are univariable and multivariable liner regression β coefficients with corresponding *P*-values. Multivariable analyses adjusted for age, sex, BMI, eGFR, serum phosphate, bicarbonate, 1-84 PTH, 25(OH)D and CRP. Skewed biochemical variables were log transformed to satisfy linear regression assumptions.

Significant values are in bold.

Liver ALP was independently associated with CRP across both CKD G1–5 and G5D (Table 4). In CKD G1–5 and CKD G5D, lower 25(OH)D and higher PTH, respectively, were also determinants of liver ALP.

**Table 4:** Determinants of liver ALP in CKD G1–5 and G5D.

	CKD G1–5				CKD G5D			
Variable	Univariable		Multivariable (*R*^2^ = 0.2145)		Univariable		Multivariable (*R*^2^ = 0.212)	
	β	*P*-value	β	*P*-value	β	*P*-value	β	*P*-value
Intercept			36.349	**<.001**			2.697	**<.001**
Age (years)	0.002	.243	−0.001	.647	0.020	.436	0.005	.140
Sex (male)	−0.008	.876	−0.015	.793	0.069	.387	0.098	.254
BMI (kg/m^2^)	0.010	.072	−0.002	.733	−0.006	.469	−0.010	.252
eGFR (ml/min/1.73 m^2^)	−0.002	**.036**	−0.001	.479				
Phosphate (mg/dl)	0.070	**.045**	0.047	.246	−0.061	**.022**	−0.045	.162
Bicarbonate (mmol/l)	−0.007	.431	0.012	.264	0.024	.089	0.018	.250
ln 1-84 PTH (pg/ml)	0.038	.110	0.018	.536	0.131	**<.001**	0.122	**.001**
ln 25(OH)D (ng/ml)	−0.156	**.003**	−0.124	**.031**	0.131	.113	0.058	.520
ln CRP (mg/l)	0.173	**<.001**	0.218	**<.001**	0.160	**.000**	0.167	**<.001**

Data are univariable and multivariable liner regression β coefficients with corresponding *P*-values. Multivariable analyses adjusted for age, sex, BMI, eGFR, serum phosphate, bicarbonate, 1-84 PTH, 25(OH)D and CRP. Skewed biochemical variables were log transformed to satisfy linear regression assumptions.

Significant values in bold.

Including GGT as a covariate did not materially change the results (Supplementary Table S5).

Bone ALP was independently associated with PTH across both CKD G1–5 and G5D (Table 5). In CKD G1–5, younger age, lower eGFR and lower 25(OH)D were also associated with higher bone ALP, while in G5D, female sex, lower BMI and lower phosphate were additional determinants.

**Table 5:** Determinants of bone ALP in CKD G1–5 and G5D.

	CKD G1-5				CKD G5D			
Variable	Univariable		Multivariable (*R*^2^ = 0.2327)		Univariable		Multivariable (*R*^2^ = 0.384)	
	β	*P*-value	β	*P*-value	β	*P*-value	β	*P*-value
Intercept			37 765	**<.001**			39 380	**<.001**
Age (years)	−0.003	.144	−0.005	**.016**	−0.106	**.001**	−0.005	.088
Sex (male)	−0.076	.183	−0.110	.077	−0.195	**.039**	−0.237	**.009**
BMI (kg/m^2^)	−0.009	.115	−0.009	.161	−0.021	**.044**	−0.027	**.004**
eGFR (ml/min/1.73 m^2^)	−0.003	**.001**	−0.003	**.019**				
Phosphate (mg/dl)	0.073	.052	0.009	.831	−0.013	.686	−0.081	**.016**
Bicarbonate (mmol/l)	−0.020	**.037**	0.005	.660	0.012	.488	−0.016	.310
ln PTH (pg/ml)	0.130	**<.001**	0.117	**<.001**	0.265	**<.001**	0.248	**<.001**
ln 25(OH)D (ng/ml)	−0.057	.313	−0.125	**.044**	0.310	**.002**	0.124	.184
ln CRP (mg/l)	−0.040	.219	−0.021	.568	0.003	.950	0.054	.265

Data are univariable and multivariable liner regression β coefficients with corresponding *P*-values. Multivariable analyses adjusted for age, sex, BMI, eGFR, serum phosphate, bicarbonate, PTH, 25(OH)D and CRP. Skewed biochemical variables were log transformed to satisfy linear regression assumptions.

Significant values in bold.

### ALP fractions and mortality

Survival analyses were performed on the longitudinal HD subcohort (*n* = 82) and CKD G1–5 subcohort (*n* = 321) separately. The leading cause of death was cardiovascular, representing 40% in the HD cohort and 36% in the stage G1–5 cohort, followed by infection and malignancy. The distribution of causes of death was similar in the HD cohort (Supplementary Fig. S1).

**Figure 2:**
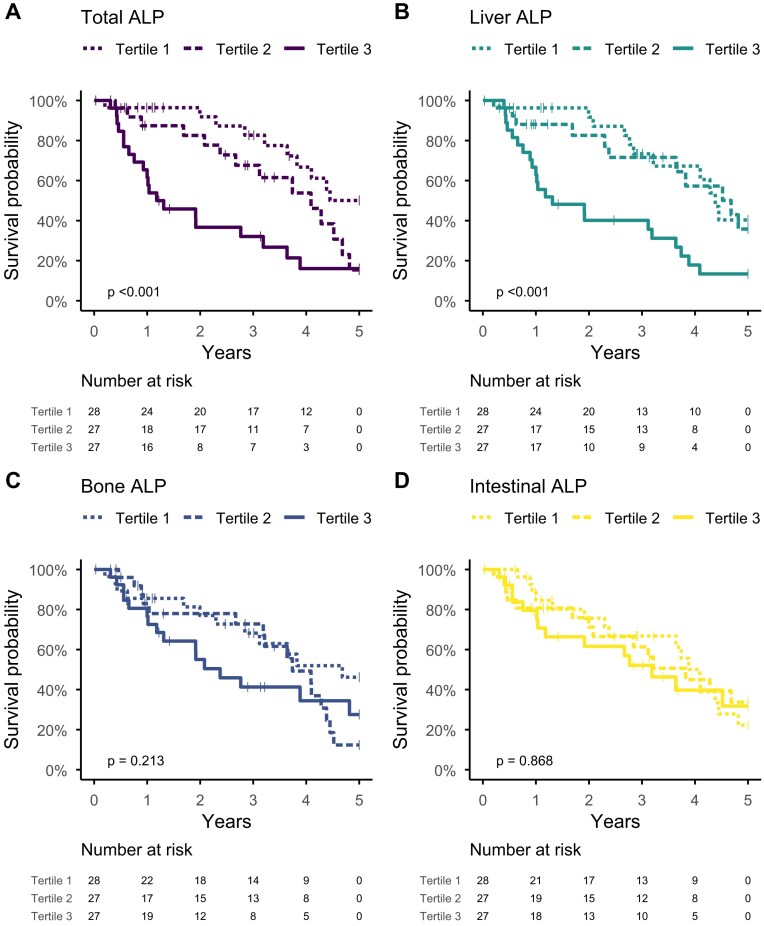
Kaplan–Meier curves of 5-year mortality across baseline **(A)** total, **(B)** liver, **(C)** bone and **(D)** intestinal ALP tertiles in 82 patients receiving HD therapy. Highest tertile of total and liver ALP was associated with a higher mortality rate.

In the HD cohort, 44 of 82 patients died over a total of 209 patient-years of follow-up (Supplementary Table S6). Patients in the highest tertiles of total or liver ALP had reduced survival, whereas bone and intestinal ALP were not associated with mortality (Fig. 2). In the Cox regression, both total and liver ALP remained independently associated with mortality after adjusting for age, sex, BMI, diabetes and cardiovascular disease (model 1). Further adjustment for PTH, CRP and ALT did not alter these associations (models 2 and 3; Table 6). Interaction terms with age and sex were not significant.

**Table 6:** Cox proportional hazard model of serum total, bone and liver ALP associations with 5-year mortality in 82 patients receiving HD therapy.

	Total ALP			Liver ALP			Bone ALP		
Model	HR^a^	95% CI	*P*-value	HR^a^	95% CI	*P*-value	HR^a^	95% CI	*P*-value
Crude	1.62	1.19–2.22	**.002**	1.54	1.19–1.99	**<.001**	1.33	0.94–1.87	.11
Model 1	1.61	1.12–2.31	**.010**	1.51	1.11–2.06	**.009**	1.23	0.83–1.83	.3
Model 2	2.35	1.40–3.96	**.001**	1.89	1.25–2.84	**.002**	1.37	0.78–2.40	.3
Model 3	2.63	1.56–4.42	**<.001**	2.17	1.43–3.30	**<.001**	1.37	0.78–2.40	.3

Model 1: adjusted for age, sex, BMI, diabetes mellitus, prior cardiovascular disease, dialysis vintage. Model 2: adjusted for PTH and CRP in addition to model 1. Model 3: adjusted of ALT in addition to model 2 (n = 79).

Significant values in bold.

a HRs are reported per doubling of total, bone or intestinal ALP.

**Figure 3:**
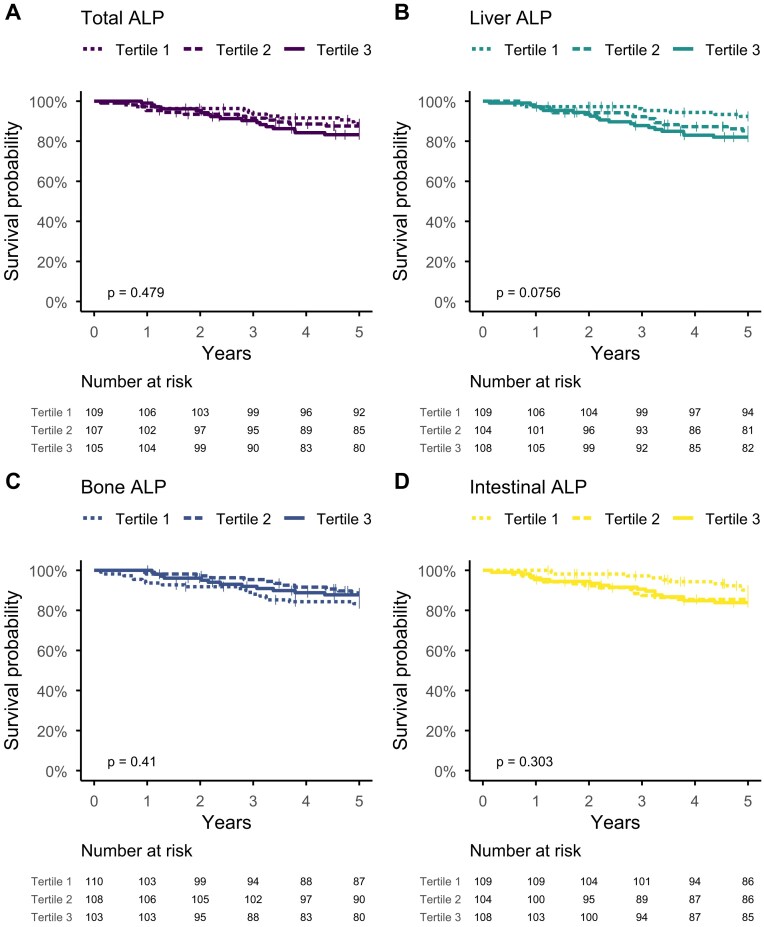
Kaplan–Meier curves of 5-year mortality across baseline **(A)** total, **(B)** liver, **(C)** bone and **(D)** intestinal ALP tertiles in 321 patients with CKD G1–5. Highest tertile of total and liver ALP was associated with a higher mortality rate.

In the CKD G1–5 cohort, 42 of 321 patients died over a total of 1450 patient-years of follow-up. Tertiles of total ALP or any of the fractions based on Kaplan–Meier analyses did not show differences in survival (Fig. 3). In the Cox regression, total ALP was not associated with mortality in unadjusted analyses, showing a modest association when adjusted for demographics, comorbidities and eGFR [hazard ratio (HR) 1.83 (95% CI 1.05–3.19)] but not after further adjustment for CRP (Table 7). Liver ALP was associated with mortality in univariate analysis and remained significantly associated after adjustment for demographics, comorbidities and eGFR [HR 1.68 (95% CI 1.06–2.67)]; however, this association also lost significance after inclusion of CRP. Bone ALP was not associated with mortality in unadjusted or adjusted models.

**Table 7:** Cox proportional hazard model of serum total, bone and liver ALP associations with 5-year mortality in 321 patients with CKD G1–5.

	Total ALP			Liver ALP			Bone ALP		
Model	HR^a^	95% CI	*P*-value	HR^a^	95% CI	*P*-value	HR^a^	95% CI	*P*-value
Crude	1.40	0.86–2.30	.2	1.58	1.05–2.37	**.028**	0.81	0.54–1.21	.3
Model 1	1.83	1.05–3.19	**.033**	1.68	1.06–.67	**.028**	1.36	0.80–2.30	.3
Model 2	1.67	0.91–3.06	.10	1.31	0.77–2.23	.3	1.61	0.94–2.73	.080

Model 1 adjusted for age, sex, BMI, eGFR, diabetes mellitus, prior cardiovascular disease (*n* = 309). Model 2 adjusted for CRP in addition to model 1.

Significant values in bold.

a HRs are reported per doubling of total, bone or intestinal ALP.

In a sensitivity analysis, we pooled both cohorts and included eGFR and dialysis vintage as subgroup variables (respectively for patients with CKD G1–5 and CKD 5D). This analysis showed consistent association between total and liver ALP with all-cause mortality (Supplementary Table S7, Supplementary Fig. S2), as opposed to bone ALP.

## DISCUSSION

This study comprehensively examined liver, bone and intestinal ALP fractions across the full spectrum of CKD, including clinical and biochemical determinants and prognostic relevance for mortality. Key findings were as follows: serum total ALP activity increased progressively with declining kidney function, driven by elevations of all three fractions; while bone ALP associated with parameters of mineral metabolism, liver ALP associated strongly with inflammation; and high liver ALP levels associated with mortality in CKD and HD patients, independent of traditional risk factors.

In agreement with previous reports [6, 14, 36, 37], we observed a stepwise increase in total ALP levels with declining kidney function, with the steepest rise after dialysis initiation. This increase has traditionally been attributed to elevations in bone ALP, particularly among dialysis patients [38]. Our data demonstrate that besides bone ALP, liver and intestinal ALP also contribute to the increase in total ALP along the progression of CKD. Circulating liver ALP activity levels were already increased in early-stage CKD, to reach levels in stage G5D that are two-fold higher than in healthy controls. Across all stages, liver ALP exceeded bone ALP activity. This finding is consistent with earlier electrophoretic studies in non-CKD individuals [19] but differs from studies in CKD using HPLC [22, 27]. Case mix and analytical differences, such as the use of different buffers, may account for the latter discrepancy [39]. The increase in intestinal ALP in dialysis patients was consistent with prior reports [28, 40].

A better understanding of the biological drivers of ALP, including the separate fractions, may identify new therapeutic targets. In our study, total ALP was consistently associated with both hyperparathyroidism and inflammation. Correlation and regression analysis identified PTH and CRP as major determinants of the bone and liver fraction of ALP, respectively. Different fractions of ALP may thus capture distinct biological processes in CKD, i.e. hyperparathyroidism versus inflammation.

The link between total ALP, via bone ALP, and PTH are well established and underpin the use of ALP as a screening marker of bone turnover in CKD [7, 41, 42]. Likewise, the link between total ALP and CRP has been reported in both CKD [9] and the general population [11, 43, 44]. Filipowicz *et al*. [10] reported an association between calculated non-skeletal ALP and CRP. We confirm this association specifically for the liver fraction. Inflammation may increase circulating ALP levels both by impairing ALP clearance and by upregulating ALP expression. Under physiological conditions, circulating ALP is cleared predominantly through hepatic uptake and intracellular degradation, which requires desialylation by endogenous neuraminidases such as NEU-4 to enable recognition by asialoglycoprotein receptors. Inflammation has been shown to downregulate neuraminidase activity, thereby reducing ALP desialylation and potentially reducing hepatic clearance [45]. In addition, liver and intestinal ALP can be upregulated in response to inflammatory stimuli as part of a mucosal defence mechanism, including the detoxification of lipopolysaccharide and degradation of extracellular adenosine triphosphate [46–50].

We also observed an independent association between PTH and liver ALP, at least in patients with kidney failure. While this may partly reflect analytical bias, related to the imperfect separation of bone and liver fractions of ALP, experimental data also suggest a biological link between PTH and hepatic metabolism [51, 52] and between PTH and systemic inflammation [53, 54]. Further, an inverse association was found between 25(OH)D levels and liver ALP in patients with CKD. Vitamin D restriction increases intestinal ALP activity and serum endotoxin concentration, increasing the risk of metabolic endotoxemia in rats [55]. Thus low vitamin D status may contribute to upregulation of liver ALP through enhanced endotoxin exposure, a hypothesis that deserves further exploration.

We found that patients treated with PD had significantly higher levels of intestinal ALP compared with those on HD. The underlying mechanisms are not entirely clear but might be related to differences in the gut microbiome [56] or to peritoneal irritation by the dialysate.

In agreement with previous studies in the general population [4, 5] and in patients with CKD [6, 12–14, 36, 37, 57], we demonstrated an independent association of higher total ALP with increased mortality in patients on HD. Importantly, we identified liver ALP, rather than bone ALP, as the fraction driving this association. In patients with CKD stage G1–5, associations between total and liver ALP and mortality were less consistent but followed the same direction. This observation is consistent with findings in the general population [10] but apparently contrasts with data reported by Drechsler *et al*. [38]. These investigators reported that bone ALP, measured by an immunometric assay, was more strongly associated with short-term mortality in patients treated with HD. High ALP has been associated with an elevated vascular calcification propensity and cardiovascular risk [7, 8, 58–60]. It is worth noting that bone ALP immunoassays can show up to 20% cross-reactivity with liver ALP, particularly at higher concentrations, and isoform separation techniques such as the electrophoretic approach used in our study may better distinguish their respective contributions [61, 62]. Although our cohort was too small to examine cause-specific mortality or distinguish short- and long-term effects, we observed a robust association between liver ALP and mortality. Elevated ALP in CKD may thus signal pathological processes beyond high bone turnover alone. Potential pathways include increased endotoxin exposure [63, 64], fluid overload, a contribution by activated leucocytes and cardiovascular calcifying, fibrotic or inflammatory processes [17], all of which warrant further evaluation.

Strengths of this study include the large, well-characterized cohort spanning the full CKD spectrum and the use of electrophoresis to separate the major circulating ALP fractions. This approach allowed us to investigate not only bone ALP but also liver and intestinal ALP.

Several limitations should be acknowledged. The cross-sectional design precludes causal inference, and residual confounding cannot be excluded. Blood samples were not strictly fasting, raising the possibility of overestimating intestinal ALP [65], although this effect is likely small in our population, as a typical Belgian breakfast is low in fat. Liver function tests such as transaminases and GGT were available only in a subset of patients. To minimize confounding, patients with a history of liver disease were excluded, and sensitivity analyses in patients with available GGT or AST data confirmed the results.

A final limitation is the absence of a universally accepted gold standard for quantifying ALP isoenzymes and isoforms. Immunoassays, electrophoresis and HPLC each have strengths, but they are validated within their own methodological framework, and results are not directly interchangeable. Analytical variability is substantial not only between different techniques but also within assays of the same type (e.g. across immunoassays or electrophoretic protocols), reflecting differences in analytical specificity and cross-reactivity between or imperfect separation of isoforms [62, 66, 67]. Our method of agarose gel electrophoresis, optimized for separating liver and bone ALP fractions and more sensitive to intestinal ALP than traditional polyacrylamide electrophoresis, represents a reasonable compromise. Our method also yielded results that are comparable to other electrophoretic methods [68]. Still, some overlap between bone and liver fractions is possible at high total ALP activity, and resolution for minor isoforms remains limited. Identifying determinants of circulating intestinal ALP may require more specific immunological methods [7]. As liver and bone ALP are highly glycosylated isoforms of the same protein, a better characterization of the specific glycosylation patterns may enable the design of more specific immunological or non-immunological assays [69]. Future progress will require harmonization of techniques through cross-validation of immunoassays, electrophoresis and HPLC in the same samples, ideally leading to reference methods that improve comparability across studies. Reliable immunoassays for liver and intestinal ALP are not yet available and the development of reproducible high-throughput immunoassays would enable analyses in much larger cohorts.

Our findings confer several important clinical messages. First, the current Kidney Disease: Improving Global Outcomes clinical practice guidelines on CKD-MBD recommend annual measurement of total ALP in patients with CKD G4–5 as an adjunct test when monitoring medical therapy or to assess for increased bone turnover in the presence of secondary hyperparathyroidism. Clinicians adopting this strategy should be aware of the potential bias by inflammation in addition to liver dysfunction per se when considering total ALP as a proxy for bone ALP. Second, high total ALP may signal increased mortality risk not only through disturbances in mineral metabolism, but also through an underlying inflammatory state, with liver ALP emerging as a key fraction in this pathway.

In conclusion, increases in circulating total ALP observed in CKD reflect contributions from liver, bone and intestinal fractions. While bone ALP is linked to mineral metabolism, liver ALP reflects systemic inflammation and was found to be the fraction most strongly associated with mortality in patients treated with HD. Further studies should elucidate the mechanisms driving liver ALP and assess its potential as a therapeutic target in patients with CKD.

## Supplementary Material

sfag078_Supplemental_Files

## Data Availability

The data underlying this article will be shared upon reasonable request to the corresponding author. Due to privacy and ethical restrictions, individual participant data are not publicly available.
