# Supplementary material for: Liver fraction of circulating alkaline phosphatase is elevated in chronic kidney disease and associates with mortality in patients treated with haemodialysis
Source: Clin Kidney J. 2026 Mar 11;19(4):sfag078. doi: 10.1093/ckj/sfag078 (PMC13103662; doi:10.1093/ckj/sfag078)
Supplement: sfag078_Supplemental_Files [file sfag078_supplemental_files.zip › Supplementary Tables_CKJ__ALPfractions.docx]

**Supplementary Table S1.** Patient characteristics across stages of CKD

| Characteristic | | N | Overall | Healthy | CKD G1-2 | CKD G3 | CKD G4-5 | CKD G5D | p-value |
| --- | --- | --- | --- | --- | --- | --- | --- | --- | --- |
|  |  |  | N = 544 | N = 21 | N = 90 | N = 100 | N = 139 | N = 194 |  |
| Total ALP (U/L) | | 544 | 81 (60, 111) | 51 (40, 54) | 65 (50, 82) | 73 (61, 100) | 77 (59, 101) | 112 (79, 146) | **<0.001** |
| Bone ALP (U/L) | | 544 | 24 (17, 37) | 18 (14, 21) | 19 (13, 25) | 20 (15, 29) | 22 (16, 33) | 34 (23, 52) | **<0.001** |
| Liver ALP (U/L) | | 544 | 48 (33, 66) | 26 (21, 35) | 41 (29, 53) | 50 (32, 64) | 48 (32, 63) | 56 (39, 79) | **<0.001** |
| Intestinal ALP (U/L) | | 544 | 0 (0, 12) | 0 (0, 4) | 0 (0, 4) | 0 (0, 7) | 0 (0, 8) | 9 (0, 21) | **<0.001** |
| Demographics |  |  |  |  |  |  |  |  |  |
| Age (years) | | 544 | 62 (49, 73) | 48 (29, 52) | 46 (38, 56) | 68 (57, 76) | 68 (56, 76) | 64 (52, 72) | **<0.001** |
| Sex (male, %) | | 544 | 318 (58%) | 5 (24%) | 45 (50%) | 67 (67%) | 76 (55%) | 125 (64%) | **<0.001** |
| BMI (kg/m²) | | 523 | 25.3 (22.8, 28.9) | 22.9 (21.3, 23.8) | 25.7 (22.6, 29.4) | 26.1 (23.7, 30.0) | 25.9 (22.5, 29.5) | 24.7 (22.6, 27.9) | **<0.001** |
| Diabetes mellitus (%) | | 544 | 125 (24%) | 0 0%) | 11 (12%) | 25 (25%) | 28 (20%) | 61 (31%) | **0.002** |
| Prevalent CVD (%) | | 544 | 179 (34%) | 0 (0%) | 7 (7.8%) | 38 (38%) | 57 (41%) | 77 (40%) | **<0.001** |
| Malignancy (%) | | 544 | 55 (11%) | 0 (0%) | 1 (1.1%) | 14 (14%) | 16 (12%) | 24 (12%) | **0.003** |
| ADPKD (%) | | 544 | 57 (11%) | 0 (0%) | 10 (11%) | 10 (10%) | 12 (8.6%) | 25 (13%) | 0.7 |
| Biochemistry |  |  |  |  |  |  |  |  |  |
| Creatinine (mg/dL) | | 534 | 2.65 (1.40, 6.39) | 0.85 (0.72, 0.90) | 0.92 (0.77, 1.04) | 1.58 (1.40, 1.81) | 2.92 (2.25, 3.86) | 7.51 (5.82, 9.10) | **<0.001** |
| eGFR (mL/min/1.73m²) | | 534 | 22 (8, 48) | 98 (89, 110) | 88 (70, 106) | 41 (35, 48) | 20 (14, 25) | 6 (5, 8) | **<0.001** |
| Urea (mg/dL) | | 533 | 86 (50, 123) | 29 (27, 36) | 35 (27, 40) | 63 (51, 77) | 114 (91, 147) | 115 (86, 138) | **<0.001** |
| Calcium (mg/dL) | | 538 | 9.30 (9.00, 9.60) | 9.35 (9.09, 9.52) | 9.20 (8.90, 9.50) | 9.30 (9.00, 9.50) | 9.20 (8.90, 9.50) | 9.40 (9.00, 9.90) | **0.004** |
| Phosphate (mg/dL) | | 537 | 3.60 (3.00, 4.20) | 3.20 (2.89, 3.69) | 3.00 (2.60, 3.40) | 3.20 (2.75, 3.50) | 3.80 (3.30, 4.10) | 4.40 (3.60, 5.48) | **<0.001** |
| Bicarbonate (mmol/L) | | 526 | 24.70 (23.00, 26.70) | 24.75 (23.50, 27.50) | 26.20 (24.60, 27.80) | 25.25 (23.55, 27.40) | 23.55 (21.50, 25.20) | 24.45 (23.15, 26.95) | **<0.001** |
| 1-84 PTH (pg/mL) | | 498 | 46 (21, 112) | NA | 13 (4, 20) | 25 (15, 37) | 61 (37, 109) | 110 (55, 226) | **<0.001** |
| 25-(OH)D (ng/mL) | | 417 | 30 (20, 40) | NA | 23 (17, 36) | 26 (18, 32) | 27 (19, 38) | 39 (30, 52) | **<0.001** |
| Urea (24-hour urine) (g/day) | | 260 | 18 (13, 23) | NA | 22 (16, 28) | 21 (16, 27) | 18 (14, 22) | 2 (1, 3) | **<0.001** |
| Proteinuria (24-hour urine) (g/day) | | 297 | 0.26 (0.11, 0.72) | NA | 0.16 (0.10, 0.39) | 0.16 (0.11, 0.60) | 0.47 (0.21, 1.06) | 0.27 (0.18, 0.62) | **<0.001** |
| Albumin (g/L) | | 531 | 44.4 (41.8, 46.9) | 47.8 (44.9, 49.0) | 45.8 (43.2, 47.9) | 45.4 (43.8, 46.9) | 44.9 (42.9, 47.1) | 42.0 (39.1, 45.0) | **<0.001** |
| GGT (U/L) | | 387 | 22 (17, 36) | NA | 24 (16, 38) | 24 (18, 43) | 22 (17, 32) | 23 (16, 36) | 0.5 |
| AST (U/L) | | 451 | 21 (17, 26) | NA | 23 (21, 29) | 25 (21, 28) | 22 (18, 27) | 17 (14, 22) | **<0.001** |
| ALT (U/L) | | 450 | 19 (14, 25) | NA | 23 (19, 33) | 22 (18, 29) | 18 (14, 23) | 16 (12, 22) | **<0.001** |
| Bilirubin (mg/dL) | | 420 | 0.50 (0.30, 0.60) | NA | 0.55 (0.40, 0.70) | 0.40 (0.40, 0.60) | 0.40 (0.30, 0.60) | 0.45 (0.30, 0.60) | **0.005** |
| Hemoglobin (g/dL) | | 533 | 12.60 (11.60, 14.00) | 13.90 (12.90, 14.90) | 14.50 (13.50, 15.30) | 13.60 (12.40, 14.55) | 12.20 (11.10, 13.00) | 11.90 (11.20, 12.70) | **<0.001** |
| WBC (10^9/L) | | 508 | 6.60 (5.29, 7.90) | NA | 6.20 (5.20, 7.90) | 6.70 (5.40, 7.90) | 7.00 (5.30, 8.20) | 6.20 (5.28, 7.70) | 0.5 |
| CRP (mg/L) | | 486 | 3 (1, 6) | NA | 1 (1, 3) | 2 (1, 5) | 2 (1, 6) | 4 (2, 8) | **<0.001** |
| Cholesterol (mg/dL) | | 500 | 170 (147, 193) | NA | 178 (161, 201) | 173 (154, 191) | 172 (149, 195) | 161 (138, 186) | **<0.001** |
| HDL (mg/dL) | | 499 | 49 (41, 63) | NA | 58 (44, 68) | 50 (40, 62) | 48 (41, 63) | 48 (39, 62) | **0.008** |
| LDL (mg/dL) | | 490 | 87 (66, 104) | NA | 95 (75, 112) | 88 (66, 103) | 87 (68, 105) | 77 (56, 99) | **<0.001** |
| Triglycerides (mg/dL) | | 499 | 132 (90, 188) | NA | 113 (78, 171) | 134 (99, 207) | 126 (91, 188) | 137 (98, 194) | 0.091 |
| Free iron (µg/dL) | | 502 | 74 (56, 95) | NA (NA, NA) | 100 (71, 126) | 78 (65, 93) | 79 (63, 94) | 62 (46, 77) | **<0.001** |
| Transferrin (g/L) | | 501 | 2.31 (2.00, 2.60) | NA (NA, NA) | 2.56 (2.39, 2.85) | 2.48 (2.19, 2.70) | 2.37 (2.14, 2.71) | 1.97 (1.73, 2.29) | **<0.001** |
| Transferrin saturation (%) | | 501 | 22 (18, 30) | NA (NA, NA) | 27 (20, 34) | 22 (19, 27) | 22 (18, 29) | 21 (17, 29) | **0.017** |
| Ferritin (ng/mL) | | 501 | 186 (88, 344) | NA | 116 (64, 205) | 166 (87, 260) | 149 (67, 255) | 313 (160, 532) | **<0.001** |
| Medication | |  |  |  |  |  |  |  |  |
| Proton Pump Inhibitor (%) | | 523 | 125 (24%) | NA | 7 (7.8%) | 20 (20%) | 26 (19%) | 72 (37%) | **<0.001** |
| Oral Bicarbonate (%) | | 523 | 41 (7.8%) | NA | 0 (0%) | 2 (2.0%) | 27 (19%) | 12 (6.2%) | **<0.001** |
| Calcium Supplement (%) | | 523 | 179 (34%) | NA | 13 (14%) | 13 (13%) | 45 (32%) | 108 (56%) | **<0.001** |
| Phosphate Binder (Calcium-based) (%) | | 523 | 32 (6.1%) | NA | 0 (0%) | 0 (0%) | 1 (0.7%) | 31 (16%) | **<0.001** |
| Phosphate Binder (Non-Calcium-based) (%) | | 523 | 55 (11%) | NA | 0 (0%) | 0 (0%) | 0 (0%) | 55 (28%) | **<0.001** |
| Active Vitamin D (%) | | 544 | 100 (18%) | NA | 0 (0%) | 3 (3.0%) | 20 (14%) | 77 (40%) |  |
| Vitamin D (%) | | 523 | 117 (22%) | NA | 12 (13%) | 16 (16%) | 45 (32%) | 44 (23%) | **0.002** |
| Cinacalcet (%) | | 523 | 17 (3.3%) | NA | 0 (0%) | 0 (0%) | 0 (0%) | 17 (8.8%) | **<0.001** |
| Bisphosphonate (%) | | 523 | 18 (3.4%) | NA | 2 (2.2%) | 5 (5.0%) | 6 (4.3%) | 5 (2.6%) | 0.6 |
| Denosumab (%) | | 523 |  | NA | 0% | 0% | 0% | 0% |  |
| Potassium Binder (%) | | 523 | 9 (1.7%) | NA | 0 (0%) | 0 (0%) | 3 (2.2%) | 6 (3.1%) | 0.13 |
| Oral Iron (%) | | 523 | 127 (24%) | NA | 2 (2.2%) | 10 (10%) | 29 (21%) | 86 (44%) | **<0.001** |
| Erythropoiesis-stimulating agent (%) | | 523 | 160 (31%) | NA | 0 (0%) | 8 (8.0%) | 40 (29%) | 112 (58%) | **<0.001** |
| Statin (%) | | 523 | 246 (47%) | NA | 28 (31%) | 63 (63%) | 86 (62%) | 69 (36%) | **<0.001** |
| ACE Inhibitor (%) | | 523 | 213 (41%) | NA | 59 (66%) | 57 (57%) | 56 (40%) | 41 (21%) | **<0.001** |
| Angiotensin Receptor Blocker (%) | | 523 | 96 (18%) | NA | 17 (19%) | 27 (27%) | 37 (27%) | 15 (7.7%) | **<0.001** |
| Beta Blocker (%) | | 523 | 207 (40%) | NA | 17 (19%) | 43 (43%) | 72 (52%) | 75 (39%) | **<0.001** |
| Loop Diuretics (%) | | 523 | 81 (15%) | NA | 1 (1.1%) | 17 (17%) | 40 (29%) | 23 (12%) | **<0.001** |
| Thiazide(-like) diuretics (%) | | 523 | 70 (13%) | NA | 16 (18%) | 26 (26%) | 23 (17%) | 5 (2.6%) | **<0.001** |
| Oral Corticosteroids (%) | | 523 | 43 (8.2%) | NA | 11 (12%) | 5 (5.0%) | 13 (9.4%) | 14 (7.2%) | 0.3 |
| Immunosuppressant (%) | | 523 | 34 (6.5%) | NA | 10 (11%) | 6 (6.0%) | 6 (4.3%) | 12 (6.2%) | 0.3 |

Data are presented as median [Q1, Q3] for continuous variables and n (%) for categorical variables. Comparisons across CKD stages were performed using Kruskal–Wallis test for continuous variables and Chi-squared or Fisher’s exact test for categorical variables.

Abbreviations: CKD = chronic kidney disease; ALP = alkaline phosphatase; PTH = parathyroid hormone; CRP = C-reactive protein; BMI = body mass index; BP = blood pressure; CVD = cardiovascular disease.

**Supplementary Table S2**. Patient characteristics across tertiles of total ALP stratified within each stage of CKD

| **Characteristic***^1^* | **Total Alkaline Phosphatase CKD stage-adjusted tertiles*** | | | **p-value***^2^* |
| --- | --- | --- | --- | --- |
|  | **Tertile 1** | **Tertile 2** | **Tertile 3** |  |
|  | N = 183 | N = 181 | N = 180 |  |
| **Age (years)** | 65 (52, 75) | 59 (47, 70) | 62 (49, 72) | **0.014** |
| **Sex (male, %)** | 108 (59%) | 110 (61%) | 100 (56%) | 0.6 |
| **BMI (kg/m²)** | 25.2 (22.7, 29.5) | 25.4 (22.8, 28.7) | 25.5 (23.5, 28.7) | 0.8 |
| **Systolic BP (mmHg)** | 140 (125, 150) | 135 (120, 150) | 135 (120, 150) | 0.12 |
| **Diastolic BP (mmHg)** | 80 (70, 88) | 80 (70, 86) | 78 (70, 83) | 0.3 |
| **Diabetes mellitus (%)** | 50 (28%) | 35 (20%) | 40 (23%) | 0.2 |
| **Prevalent CVD (%)** | 64 (36%) | 52 (30%) | 63 (37%) | 0.4 |
| **Malignancy (%)** | 24 (14%) | 14 (8.0%) | 17 (9.8%) | 0.2 |
| **ADPKD (%)** | 23 (13%) | 18 (10%) | 16 (9.2%) | 0.5 |
| **Dialysis Vintage (months)** | 23 (13, 38) | 36 (23, 49) | 28 (14, 48) | **0.016** |
| **Biochemistry** | | | | |
| **Creatinine (mg/dL)** | 2.82 (1.44, 6.58) | 2.55 (1.41, 5.83) | 2.62 (1.33, 6.39) | 0.8 |
| **eGFR (mL/min/1.73m²)** | 21 (7, 43) | 23 (8, 50) | 22 (8, 49) | 0.6 |
| **Urea (mg/dL)** | 88 (51, 128) | 86 (49, 124) | 79 (47, 118) | 0.3 |
| **Calcium (mg/dL)** | 9.30 (9.00, 9.60) | 9.30 (9.00, 9.70) | 9.20 (8.90, 9.50) | 0.2 |
| **Phosphate (mg/dL)** | 3.60 (3.00, 4.30) | 3.60 (2.90, 4.28) | 3.70 (3.09, 4.10) | 0.7 |
| **Bicarbonate (mmol/L)** | 24.30 (23.10, 26.60) | 24.80 (23.00, 27.10) | 24.80 (23.00, 26.60) | 0.7 |
| **1-84 PTH (pg/mL)** | 36 (17, 78) | 49 (22, 106) | 66 (23, 178) | **<0.001** |
| **25-(OH)D (ng/mL)** | 27 (19, 38) | 32 (21, 42) | 25 (16, 35) | **<0.001** |
| **GGT (U/L)** | 21 (17, 32) | 21 (16, 36) | 26 (17, 48) | **0.037** |
| **AST (U/L)** | 20 (16, 26) | 22 (17, 26) | 22 (17, 26) | 0.3 |
| **ALT (U/L)** | 18 (13, 24) | 19 (14, 25) | 19 (15, 26) | 0.11 |
| **Bilirubin (mg/dL)** | 0.40 (0.30, 0.60) | 0.40 (0.30, 0.60) | 0.50 (0.30, 0.60) | 0.8 |
| **Hemoglobin (g/dL)** | 12.30 (11.50, 13.80) | 12.70 (11.80, 14.40) | 12.80 (11.70, 14.20) | **0.017** |
| **WBC (10^9/L)** | 6.40 (5.40, 8.00) | 6.70 (5.30, 7.99) | 6.50 (5.10, 7.70) | 0.6 |
| **CRP (mg/L)** | 2 (1, 6) | 2 (1, 5) | 4 (1, 8) | **<0.001** |
| **Albumin (g/L)** | 44.4 (41.4, 46.9) | 45.3 (42.5, 47.3) | 44.1 (42.0, 46.6) | 0.052 |
| **Cholesterol (mg/dL)** | 169 (145, 194) | 170 (149, 195) | 173 (147, 189) | 0.9 |
| **HDL (mg/dL)** | 48 (40, 62) | 50 (41, 63) | 51 (41, 64) | 0.8 |
| **LDL (mg/dL)** | 86 (69, 108) | 86 (65, 106) | 87 (63, 103) | 0.4 |
| **Triglycerides (mg/dL)** | 127 (90, 172) | 136 (91, 190) | 131 (88, 191) | 0.7 |
| **Free iron (µg/dL)** | 74 (56, 95) | 77 (59, 98) | 75 (57, 96) | 0.4 |
| **Transferrin (g/L)** | 2.31 (2.00, 2.60) | 2.29 (1.93, 2.62) | 2.32 (2.01, 2.59) | 0.6 |
| **Transferrin saturation (%)** | 22 (18, 30) | 23 (19, 31) | 23 (19, 29) | 0.3 |
| **Ferritin (ng/mL)** | 194 (96, 340) | 195 (90, 365) | 159 (73, 313) | 0.2 |
| **Medications** | | | | |
| **Proton Pump Inhibitor (%)** | 38 (22%) | 48 (28%) | 39 (23%) | 0.4 |
| **Oral Bicarbonate (%)** | 20 (11%) | 8 (4.6%) | 13 (7.5%) | 0.061 |
| **Calcium Supplement (%)** | 72 (41%) | 51 (29%) | 56 (32%) | 0.060 |
| **Phosphate Binder (Calcium-based) (%)** | 10 (5.7%) | 8 (4.6%) | 14 (8.1%) | 0.4 |
| **Phosphate Binder (Non-Calcium-based) (%)** | 12 (6.8%) | 25 (14%) | 18 (10%) | 0.071 |
| **Active Vitamin D Medication (%)** | 32 (17%) | 34 (19%) | 34 (19%) | >0.9 |
| **Vitamin D Medication (%)** | 43 (24%) | 35 (20%) | 39 (23%) | 0.6 |
| **Bisphosphonate (%)** | 8 (4.5%) | 6 (3.4%) | 4 (2.3%) | 0.5 |
| **Cinacalcet (%)** | 4 (2.3%) | 7 (4.0%) | 6 (3.5%) | 0.6 |
| **Potassium Binder (%)** | 3 (1.7%) | 2 (1.1%) | 4 (2.3%) | 0.7 |
| **Oral Iron (%)** | 46 (26%) | 44 (25%) | 37 (21%) | 0.5 |
| **Erythropoiesis-stimulating agent (%)** | 64 (36%) | 45 (26%) | 51 (29%) | 0.10 |
| **Statin (%)** | 77 (44%) | 80 (46%) | 89 (51%) | 0.3 |
| **ACE Inhibitor (%)** | 67 (38%) | 73 (42%) | 73 (42%) | 0.7 |
| **Angiotensin Receptor Blocker (%)** | 31 (18%) | 36 (21%) | 29 (17%) | 0.6 |
| **Beta Blocker (%)** | 69 (39%) | 59 (34%) | 79 (46%) | 0.081 |
| **Loop Diuretics (%)** | 32 (18%) | 23 (13%) | 26 (15%) | 0.4 |
| **Thiazide(-like) diuretics (%)** | 22 (13%) | 26 (15%) | 22 (13%) | 0.8 |
| **Oral Corticosteroids (%)** | 13 (7.4%) | 15 (8.6%) | 15 (8.7%) | 0.9 |
| **Immunosuppressant (%)** | 12 (6.8%) | 7 (4.0%) | 15 (8.7%) | 0.2 |

* Total alkaline phosphatase tertiles were stratified within each CKD stage and then combined across stages to create CKD-adjusted tertile groups for comparative analysis.

Data are presented as median [Q1, Q3] for continuous variables and n (%) for categorical variables.Comparisons across ALP tertiles within each CKD stage were performed using Kruskal–Wallis test for continuous variables and Chi-squared or Fisher’s exact test for categorical variables.

Abbreviations: ALP = alkaline phosphatase; CKD = chronic kidney disease; PTH = parathyroid hormone; CRP = C-reactive protein.

**Supplementary Table S3.** Univariable regressions in CKD stage G1-5

|  | Total ALP | | | Bone ALP | | | Liver ALP | | | Intestinal ALP* | |
| --- | --- | --- | --- | --- | --- | --- | --- | --- | --- | --- | --- |
| Variable | Beta | Pearson R | P-Value | Beta | Pearson R | P-Value | Beta | Pearson R | P-Value | Beta | P-Value |
| Total ALP (U/L) |  |  |  | 0.293 | 0.681 | **<0.0001** | 0.688 | 0.892 | **<0.0001** | 0.000 | 0.8793 |
| Bone ALP (U/L) | 1.583 | 0.681 | **<0.0001** |  |  |  | 0.582 | 0.325 | **<0.0001** | −0.017 | 0.0585 |
| Liver ALP (U/L) | 1.158 | 0.892 | **<0.0001** | 0.182 | 0.325 | **<0.0001** |  |  |  | −0.012 | **0.0180** |
| Intestinal ALP (U/L) | 0.580 | 0.105 | 0.0563 | 0.003 | 0.001 | 0.9803 | −0.423 | −0.100 | 0.0713 |  |  |
| Age (years) | 0.083 | 0.036 | 0.5171 | −0.071 | −0.071 | 0.2018 | 0.107 | 0.060 | 0.2809 | 0.019 | **0.0090** |
| Sex (male, %) | −3.147 | −0.042 | 0.4521 | −2.610 | −0.080 | 0.1468 | −1.601 | −0.027 | 0.6197 | 0.277 | 0.2282 |
| BMI (kg/m²) | 0.218 | 0.029 | 0.6080 | −0.287 | −0.089 | 0.1161 | 0.481 | 0.084 | 0.1424 | 0.036 | 0.1135 |
| Diabetes mellitus (%) | −6.599 | −0.070 | 0.2069 | −2.567 | −0.063 | 0.2541 | −5.901 | −0.081 | 0.1428 | 0.786 | **0.0053** |
| Prevalent CVD (%) | 7.029 | 0.087 | 0.1160 | 1.216 | 0.035 | 0.5282 | 4.884 | 0.078 | 0.1565 | 0.296 | 0.2222 |
| Malignancy (%) | 17.839 | 0.139 | **0.0115** | 5.342 | 0.097 | 0.0794 | 10.846 | 0.110 | **0.0465** | 0.701 | 0.0651 |
| ADPKD (%) | −2.634 | −0.021 | 0.7064 | −6.306 | −0.116 | **0.0355** | 3.146 | 0.032 | 0.5592 | −0.080 | 0.8349 |
| Creatinine (mg/dL) | 2.847 | 0.104 | 0.0595 | 1.707 | 0.145 | **0.0085** | 0.975 | 0.046 | 0.4040 | 0.088 | 0.2842 |
| eGFR (mL/min/1.73m²) | −0.183 | −0.170 | **0.0020** | −0.076 | −0.164 | **0.0029** | −0.102 | −0.123 | **0.0258** | −0.004 | 0.2930 |
| Urea (mg/dL) | 0.020 | 0.027 | 0.6244 | 0.022 | 0.069 | 0.2108 | −0.003 | −0.005 | 0.9216 | 0.001 | 0.6005 |
| Calcium (mg/dL) | −4.929 | −0.062 | 0.2628 | −2.706 | −0.079 | 0.1532 | −1.887 | −0.031 | 0.5785 | 0.002 | 0.9937 |
| Phosphate (mg/dL) | 6.602 | 0.132 | **0.0170** | 2.571 | 0.119 | **0.0313** | 3.557 | 0.092 | 0.0951 | 0.101 | 0.5037 |
| Bicarbonate (mmol/L) | −1.774 | −0.138 | **0.0124** | −0.630 | −0.114 | **0.0394** | −0.956 | −0.096 | 0.0814 | −0.023 | 0.5585 |
| PTH (pg/mL) | 0.181 | 0.275 | **<0.0001** | 0.128 | 0.447 | **<0.0001** | 0.049 | 0.098 | 0.0876 | 0.001 | 0.5370 |
| Vitamin D Level (ng/mL) | −0.312 | −0.116 | **0.0397** | −0.047 | −0.041 | 0.4724 | −0.252 | −0.121 | **0.0324** | 0.001 | 0.8944 |
| Urea (24-hour) (g/day) | −1.091 | −0.187 | **0.0049** | −0.319 | −0.131 | 0.0504 | −0.768 | −0.169 | **0.0113** | 0.003 | 0.8926 |
| Proteinuria (24-hour) (g/day) | 1.798 | 0.042 | 0.4941 | 0.985 | 0.055 | 0.3677 | −0.168 | −0.005 | 0.9348 | 0.255 | **0.0621** |
| GGT (U/L) | 0.202 | 0.291 | **<0.0001** | −0.012 | −0.040 | 0.5186 | 0.213 | 0.392 | **<0.0001** | −0.003 | 0.3578 |
| AST (U/L) | 0.498 | 0.113 | 0.0647 | −0.028 | −0.015 | 0.8063 | 0.487 | 0.141 | **0.0205** | 0.001 | 0.9716 |
| ALT (U/L) | 0.359 | 0.112 | 0.0667 | 0.031 | 0.023 | 0.7118 | 0.285 | 0.114 | 0.0625 | 0.000 | 0.9947 |
| Bilirubin (mg/dL) | −1.832 | −0.011 | 0.8539 | −2.224 | −0.040 | 0.5163 | 3.184 | 0.024 | 0.7049 | −0.621 | 0.3010 |
| CRP (mg/L) | 0.321 | 0.134 | **0.0212** | −0.087 | −0.083 | 0.1526 | 0.413 | 0.224 | **0.0001** | 0.008 | 0.3032 |
| Hemoglobin (g/dL) | −1.115 | −0.054 | 0.3265 | −0.158 | −0.018 | 0.7472 | −0.758 | −0.048 | 0.3869 | −0.060 | 0.3332 |
| WBC (10^9/L) | −0.102 | −0.007 | 0.8995 | −0.417 | −0.066 | 0.2310 | 0.115 | 0.010 | 0.8538 | 0.135 | **0.0073** |
| Albumin (g/L) | −1.711 | −0.146 | **0.0081** | −0.082 | −0.016 | 0.7707 | −1.504 | −0.167 | **0.0025** | −0.033 | 0.3549 |
| Cholesterol (mg/dL) | −0.034 | −0.031 | 0.5832 | 0.011 | 0.023 | 0.6810 | −0.019 | −0.022 | 0.6932 | −0.010 | **0.0047** |
| HDL (mg/dL) | 0.070 | 0.033 | 0.5536 | 0.108 | 0.120 | **0.0325** | −0.027 | −0.016 | 0.7717 | −0.008 | 0.2172 |
| LDL (mg/dL) | −0.064 | −0.050 | 0.3781 | −0.016 | −0.029 | 0.6031 | −0.020 | −0.020 | 0.7197 | −0.011 | **0.0102** |
| Triglycerides (mg/dL) | −0.006 | −0.013 | 0.8204 | −0.005 | −0.025 | 0.6595 | 0.001 | 0.004 | 0.9392 | 0.000 | 0.9294 |
| Ferritin (ng/mL) | −0.021 | −0.106 | 0.0594 | −0.008 | −0.099 | 0.0784 | −0.014 | −0.097 | 0.0868 | 0.001 | 0.3115 |

Data are univariable linear regression β coefficients with corresponding p-values.
* Intestinal ALP was dichotomized (present or absent) and logistic regression reported.
Abbreviations: ALP = alkaline phosphatase; PTH = parathyroid hormone; CRP = C-reactive protein; eGFR = estimated glomerular filtration rate; BMI = body mass index.

**Supplementary Table S4.** Univariable regression in CKD G5D

|  | Total ALP | | | Bone ALP | | | Liver ALP | | | Intestinal ALP* | |
| --- | --- | --- | --- | --- | --- | --- | --- | --- | --- | --- | --- |
| Variable | Beta | Pearson R | Pearson P-Value | Beta | Pearson R | Pearson P-Value | Beta | Pearson R | Pearson P-Value | Beta | P-Value |
| Total ALP (U/L) |  |  |  | 0.4892 | 0.7505 | **<0.0001** | 0.4558 | 0.7322 | **<0.0001** | 0.000 | 0.9006 |
| Bone ALP (U/L) | 11.509 | 0.7460 | **<0.0001** |  |  |  | 0.1797 | 0.1882 | **0.0086** | −0.011 | **0.0140** |
| Liver ALP (U/L) | 12.031 | 0.7447 | **<0.0001** | 0.1971 | 0.1882 | **0.0086** |  |  |  | −0.002 | 0.6383 |
| Intestinal ALP (U/L) | 0.8503 | 0.2174 | **0.0023** | −0.1850 | −0.0730 | 0.3118 | 0.0353 | 0.0146 | 0.8401 |  |  |
| Age (years) | −0.2902 | −0.0622 | 0.3885 | −0.6551 | −0.2168 | **0.0024** | 0.3477 | 0.1205 | 0.0942 | 0.019 | 0.0695 |
| Sex (male, %) | −16.3452 | −0.1152 | 0.1097 | −15.4589 | −0.1681 | **0.0191** | −0.3474 | −0.0040 | 0.9564 | 0.168 | 0.5866 |
| BMI (kg/m²) | −1.1532 | −0.0763 | 0.2929 | −0.9779 | −0.0992 | 0.1711 | −0.4455 | −0.0476 | 0.5119 | 0.010 | 0.7744 |
| Diabetes mellitus (%) | 26.517 | 0.0181 | 0.8019 | 26.279 | 0.0277 | 0.7013 | 0.4832 | 0.0053 | 0.9411 | −0.171 | 0.5909 |
| Prevalent CVD (%) | 16.398 | 0.0118 | 0.8718 | −2.8798 | −0.0319 | 0.6617 | 17.548 | 0.0203 | 0.7802 | 0.246 | 0.4237 |
| Malignancy (%) | −36.1975 | −0.1754 | **0.0144** | −22.8827 | −0.1711 | **0.0170** | −15.1760 | −0.1188 | 0.0988 | 1.179 | **0.0385** |
| ADPKD (%) | −11.1016 | −0.0548 | 0.4483 | −12.7698 | −0.0972 | 0.1777 | 25.935 | 0.0207 | 0.7749 | 0.233 | 0.6097 |
| Dialysis Vintage (months) | 0.2721 | 0.0930 | 0.3295 | −0.0408 | −0.0163 | 0.8648 | 0.1716 | 0.1118 | 0.2404 | 0.018 | 0.0972 |
| Creatinine (mg/dL) | −2.8792 | −0.1058 | 0.1494 | −0.4910 | −0.0277 | 0.7067 | −3.1956 | −0.1903 | **0.0091** | 0.036 | 0.5555 |
| eGFR (mL/min/1.73m²) | 0.9438 | 0.0563 | 0.4439 | −0.2057 | −0.0188 | 0.7980 | 15.721 | 0.1520 | **0.0378** | −0.044 | 0.2315 |
| Urea (mg/dL) | −0.0677 | −0.0380 | 0.6061 | 0.0065 | 0.0056 | 0.9400 | −0.1109 | −0.1011 | 0.1697 | 0.002 | 0.5908 |
| Calcium (mg/dL) | 19.499 | 0.0223 | 0.7594 | 0.8261 | 0.0145 | 0.8423 | 27.220 | 0.0503 | 0.4894 | 0.078 | 0.6851 |
| Phosphate (mg/dL) | −3.0673 | −0.0653 | 0.3695 | −2.2723 | −0.0742 | 0.3080 | −3.3214 | −0.1142 | 0.1156 | 0.251 | **0.0246** |
| Bicarbonate (mmol/L) | 20.839 | 0.0853 | 0.2551 | 0.8448 | 0.0527 | 0.4820 | 10.447 | 0.0691 | 0.3569 | −0.043 | 0.4312 |
| PTH (pg/mL) | 0.0831 | 0.3262 | **<0.0001** | 0.0440 | 0.2648 | **0.0002** | 0.0341 | 0.2163 | **0.0027** | 0.000 | 0.9663 |
| Vitamin D Level (ng/mL) | 0.6994 | 0.1791 | **0.0273** | 0.6790 | 0.2579 | **0.0013** | 0.0745 | 0.0308 | 0.7067 | −0.020 | **0.0404** |
| GGT (U/L) | 0.5181 | 0.2833 | **0.0018** | 0.1586 | 0.1027 | 0.2665 | 0.4133 | 0.4441 | **<0.0001** | −0.006 | 0.3393 |
| AST (U/L) | 29.808 | 0.2975 | **<0.0001** | 0.0340 | 0.0052 | 0.9448 | 26.405 | 0.4271 | **<0.0001** | 0.011 | 0.6282 |
| ALT (U/L) | 24.630 | 0.2740 | **0.0002** | 0.0843 | 0.0144 | 0.8474 | 20.669 | 0.3739 | **<0.0001** | 0.013 | 0.5200 |
| Bilirubin (mg/dL) | 306.924 | 0.0857 | 0.2814 | −17.3107 | −0.0751 | 0.3455 | 470.030 | 0.2091 | **0.0080** | −0.816 | 0.3275 |
| CRP (mg/L) | 0.8866 | 0.1745 | **0.0163** | 0.0195 | 0.0059 | 0.9356 | 0.8737 | 0.2768 | **0.0001** | −0.008 | 0.4808 |
| Hemoglobin (g/dL) | 0.5104 | 0.0101 | 0.8913 | 19.331 | 0.0583 | 0.4281 | −0.2115 | −0.0068 | 0.9268 | −0.019 | 0.8654 |
| WBC (10^9/L) | −0.3266 | −0.0091 | 0.9036 | −1.9752 | −0.0834 | 0.2642 | 15.293 | 0.0684 | 0.3600 | −0.002 | 0.9815 |
| Albumin (g/L) | 14.267 | 0.1086 | 0.1402 | 11.723 | 0.1365 | 0.0633 | 0.3376 | 0.0417 | 0.5720 | −0.004 | 0.8814 |
| Cholesterol (mg/dL) | −0.1027 | −0.0569 | 0.4434 | 0.0169 | 0.0143 | 0.8473 | −0.1200 | −0.1082 | 0.1438 | −0.005 | 0.2524 |
| HDL (mg/dL) | 0.0936 | 0.0236 | 0.7507 | 0.2758 | 0.1065 | 0.1514 | −0.1548 | −0.0637 | 0.3916 | 0.001 | 0.8990 |
| LDL (mg/dL) | −0.2022 | −0.0938 | 0.2169 | −0.0335 | −0.0239 | 0.7537 | −0.1540 | −0.1160 | 0.1264 | −0.004 | 0.3846 |
| Triglycerides (mg/dL) | −0.0045 | −0.0081 | 0.9132 | −0.0150 | −0.0413 | 0.5793 | 0.0034 | 0.0100 | 0.8934 | 0.000 | 0.7486 |
| Ferritin (ng/mL) | −0.0044 | −0.0485 | 0.5123 | −0.0027 | −0.0455 | 0.5384 | −0.0008 | −0.0139 | 0.8506 | 0.000 | 0.6823 |

Data are univariable linear regression β coefficients with corresponding p-values.
* Intestinal ALP was dichotomized (present or absent) and logistic regression reported.
Abbreviations: ALP = alkaline phosphatase; PTH = parathyroid hormone; CRP = C-reactive protein; eGFR = estimated glomerular filtration rate; BMI = body mass index.

**Supplementary table S5.** Multivariable linear regression including GGT

|  | Total ALP | | Bone ALP | | Liver ALP | |
| --- | --- | --- | --- | --- | --- | --- |
| Variable | β | P | β | P | β | P |
| Intercept | 32,092 | **<0,001** | 34,114 | **<0,001** | 24,309 | **<0,001** |
| Age (years) | -0,003 | **0,042** | -0,008 | **<0,001** | <,0012 | 0,907 |
| Sex (male, %) | -0,072 | 0,139 | -0,104 | 0,096 | -0,035 | 0,519 |
| BMI (kg/m²) | -0,006 | 0,186 | -0,016 | **0,010** | -0,005 | 0,356 |
| eGFR (mL/min/1,73m²) | -0,002 | **0,040** | -0,004 | **0,003** | -0,0018 | 0,457 |
| Phosphate (mg/dL) | 0,016 | 0,472 | -0,039 | 0,167 | 0,009 | 0,717 |
| Bicarbonate (mmol/L) | 0,021 | **0,007** | 0,012 | 0,215 | 0,022 | **0,010** |
| ln PTH (pg/mL) | 0,145 | **<0,001** | 0,215 | **<0,001** | 0,070 | **0,006** |
| ln 25(OH)D (ng/mL) | 0,016 | 0,713 | <0,0012 | 0,998 | -0,021 | 0,685 |
| ln CRP (mg/L) | 0,126 | **<0,001** | 0,049 | 0,135 | 0,175 | **<0,001** |
| ln GGT (U/L) | 0,111 | **0,004** | -0,029 | 0,557 | 0,187 | **<0,001** |

Data are multivariable linear regression β coefficients with corresponding p-values. Skewed biochemical variables were log transformed to better adhere to assumptions.

Abbreviations: ALP = alkaline phosphatase; GGT = gamma-glutamyl transferase; PTH = parathyroid hormone; CRP = C-reactive protein; eGFR = estimated glomerular filtration rate; BMI = body mass index.

**Supplementary Table S6.** Baseline characteristics of hemodialysis cohort

| Characteristic | N | Median (Q1, Q3);  n (%) |
| --- | --- | --- |
| Outcome data |  |  |
| Months of follow up | 82 | 31 (11, 49) |
| Mortality (%) | 82 | 44 (54%) |
| Kidney transplantation (%) | 82 | 24 (29%) |
| Isozymes |  |  |
| Total ALP (U/L) | 82 | 81 (66, 124) |
| Bone ALP (U/L) | 82 | 25 (19, 41) |
| Liver ALP (U/L) | 82 | 43 (32, 61) |
| Intestinal ALP (U/L) | 82 | 9 (0, 19) |
| Demographics |  |  |
| Age (years) | 82 | 72 (63, 77) |
| Sex (male, %) | 82 | 47 (57%) |
| BMI (kg/m²) | 82 | 23.7 (21.8, 26.3) |
| Diabetes mellitus (%) | 82 | 31 (38%) |
| Prevalent CVD (%) | 82 | 50 (61%) |
| Malignancy (%) | 82 | 17 (21%) |
| ADPKD (%) | 82 | 8 (9.8%) |
| Dialysis Vintage (months) | 82 | 25 (14, 50) |
| Biochemistry |  |  |
| Creatinine (mg/dL) | 82 | 7.49 (5.82, 8.80) |
| eGFR (mL/min/1.73m²) | 82 | 5.83 (4.76, 7.48) |
| Urea (mg/dL) | 82 | 118 (100, 138) |
| Calcium (mg/dL) | 82 | 9.60 (9.00, 9.90) |
| Phosphate (mg/dL) | 82 | 4.60 (3.60, 5.30) |
| Bicarbonate (mmol/L) | 77 | 23.60 (22.50, 24.70) |
| PTH (pg/mL) | 82 | 57 (30, 108) |
| Vitamin D Level (ng/mL) | 50 | 20 (14, 27) |
| AST (U/L) | 78 | 16.0 (13.0, 20.0) |
| ALT (U/L) | 79 | 14 (11, 18) |
| CRP (mg/L) | 82 | 6 (3, 9) |
| Hemoglobin (g/dL) | 82 | 11.65 (10.80, 12.40) |
| WBC (10^9/L) | 76 | 6.40 (5.25, 7.75) |
| Albumin (g/L) | 82 | 40.20 (37.80, 42.20) |
| Cholesterol (mg/dL) | 82 | 153 (130, 174) |
| HDL (mg/dL) | 82 | 53 (42, 61) |
| LDL (mg/dL) | 82 | 70 (54, 91) |
| Triglycerides (mg/dL) | 82 | 128 (96, 165) |
| Ferritin (ng/mL) | 82 | 268 (161, 448) |
| Medications |  |  |
| Proton Pump Inhibitor (%) | 82 | 33 (40%) |
| Calcium Supplement (%) | 82 | 63 (77%) |
| Phosphate Binder (Non-Calcium-based) (%) | 82 | 21 (26%) |
| Active Vitamin D Medication (%) | 82 | 33 (40%) |
| Vitamin D Medication (%) | 82 | 3 (3.7%) |
| Bisphosphonate (%) | 82 | 5 (6.1%) |
| Cinacalcet (%) | 82 | 6 (7.3%) |
| Potassium Binder (%) | 82 | 2 (2.4%) |
| Oral Iron (%) | 82 | 64 (78%) |
| Erythropoiesis-stimulating agent (%) | 82 | 73 (89%) |
| Statin (%) | 82 | 23 (28%) |
| ACE Inhibitor (%) | 82 | 11 (13%) |
| Angiotensin Receptor Blocker (%) | 82 | 3 (3.7%) |
| Beta Blocker (%) | 82 | 34 (41%) |
| Loop Diuretics (%) | 82 | 7 (8.5%) |
| Oral Corticosteroids (%) | 82 | 6 (7.3%) |
| Immunosuppressant (%) | 82 | 2 (2.4%) |

Data are presented as median [Q1, Q3] for continuous variables and n (%) for categorical variables.

Abbreviations: ALP = alkaline phosphatase; HD = hemodialysis; PTH = parathyroid hormone; CRP = C-reactive protein; BMI = body mass index; CVD = cardiovascular disease.

Supplementary Table S7 Cox proportional hazard model of serum total, bone and liver alkaline phosphatase (ALP) associations with 5-year mortality in 404 patients with CKD G1-5D

| **Overall mortality in prospective cohort (n=404)** | | | | | | | | | | | |
| --- | --- | --- | --- | --- | --- | --- | --- | --- | --- | --- | --- |
|  | | | **Total ALP** | | | **Bone ALP** | | | **Liver ALP** | | |
| **Model** | **HR*** | **95% CI** | | **P-value** | **HR*** | **95% CI** | **P-value** | **HR*** | | **95% CI** | **P-value** |
| Crude | 1.99 | 1.49, 2.65 | | <0.001 | 1.37 | 1.05, 1.78 | 0.018 | 1.67 | | 1.29, 2.16 | <0.001 |
| Model 1 | 1.66 | 1.25, 2.21 | | <0.001 | 1.30 | 0.96, 1.76 | 0.089 | 1.55 | | 1.22, 1.98 | <0.001 |
| Model 2 | 1.49 | 1.04, 2.13 | | 0.031 | 1.12 | 0.80, 1.58 | 0.5 | 1.45 | | 1.08, 1.95 | 0.014 |
| Model 3 | 1.88 | 1.23, 2.87 | | 0.003 | 1.13 | 0.78, 1.66 | 0.5 | 1.82 | | 1.28, 2.58 | <0.001 |

Model 1: adjusted for age, sex, BMI, diabetes mellitus, prior cardiovascular disease, eGFR, dialysis status, dialysis vintage
Model 2: adjusted for PTH and CRP in addition to model 1 (n=339)

Model 3: adjusted of ALT in addition to model 2 (n=279)

*Hazard ratios are reported per doubling of total, bone or intestinal ALP.

**
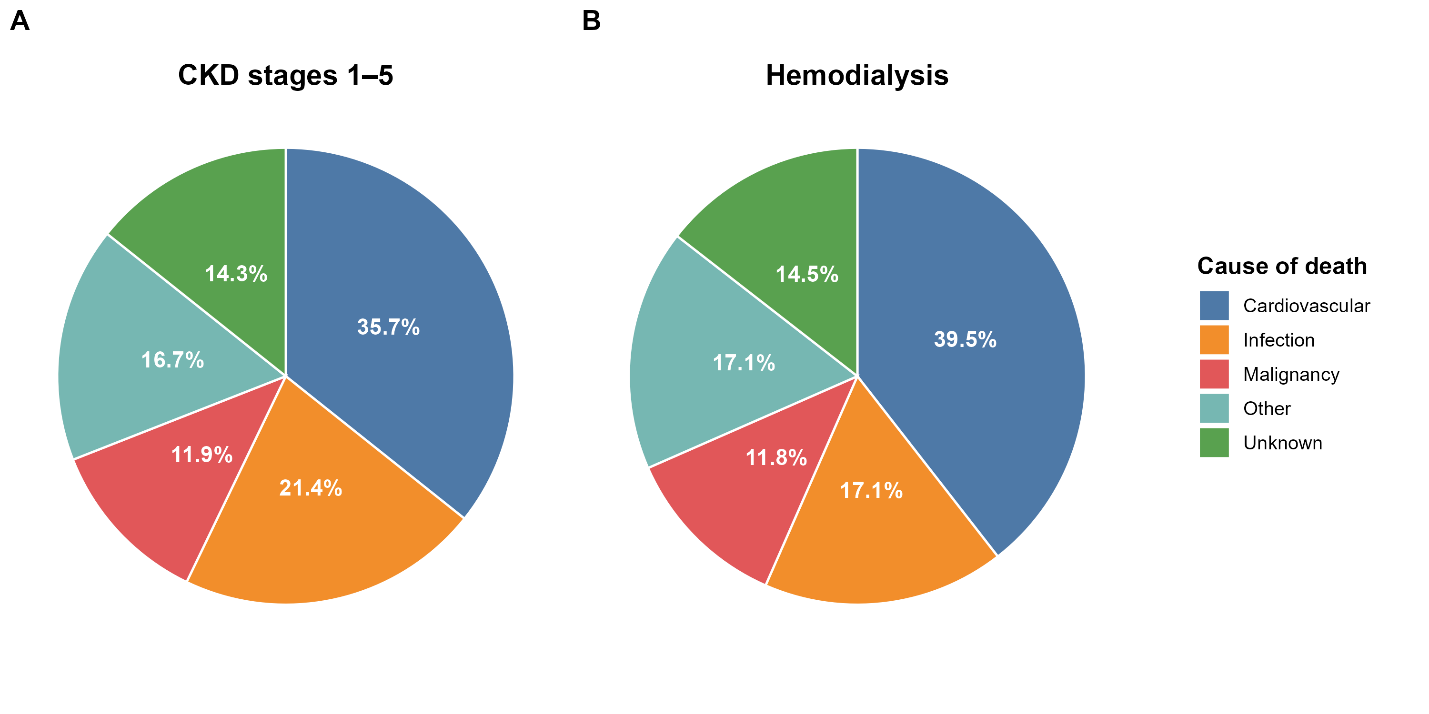
**

**Supplementary Figure S1. Causes of death in CKD G1–5 and hemodialysis patients**

Proportion of causes of death within 5 years in (A) patients with CKD G1–5 (n=42 deaths) and (B) patients on maintenance hemodialysis (n= 45 deaths). Percentages represent the share of each cause among deceased patients in each cohort.


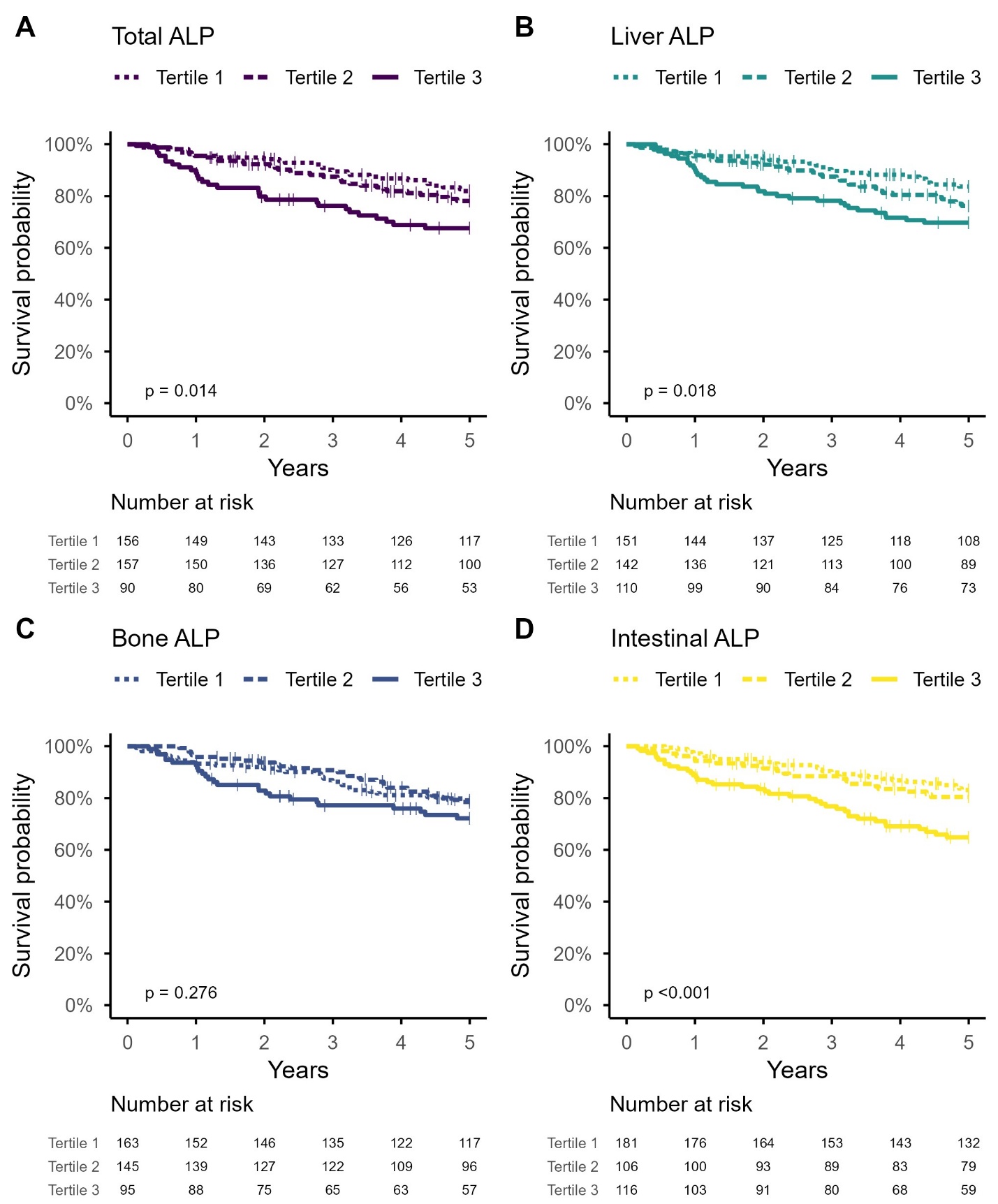


**Supplementary Figure S2. Survival in CKD G1-5D** Kaplan Meier curves of 5-year mortality across baseline (A) total, (B) Liver, (C) Bone, and (D) Intestinal alkaline phosphatase (ALP) tertiles in 404 patients with CKD G1-5 and G5D. Highest tertile of total and liver ALP was associated with higher mortality rate.
