# Supplementary figures and images for: Liver fraction of circulating alkaline phosphatase is elevated in chronic kidney disease and associates with mortality in patients treated with haemodialysis
Source: Clin Kidney J. 2026 Mar 11;19(4):sfag078. doi: 10.1093/ckj/sfag078 (PMC13103662; doi:10.1093/ckj/sfag078)

A

CKD stages 1–5

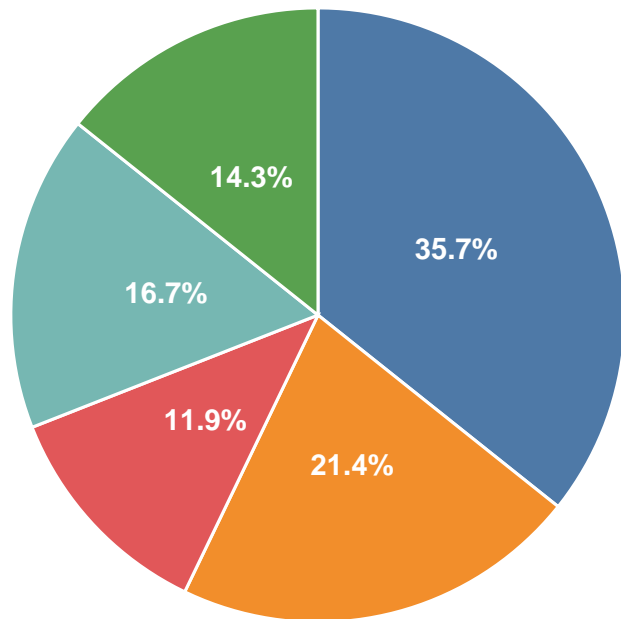

B

Cause of death

Hemodialysis

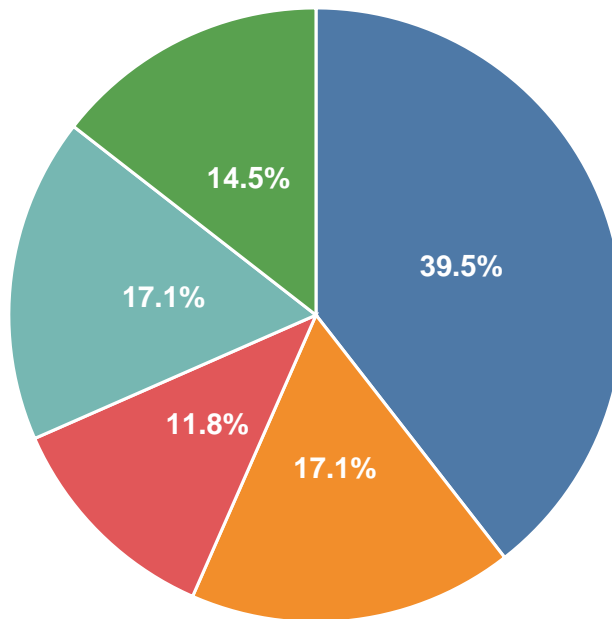

Cause of death

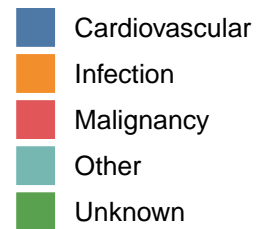

Supplement: sfag078_Supplemental_Files [file sfag078_supplemental_files.zip › Suppl_Fig_S1_CauseOfDeath.pdf]

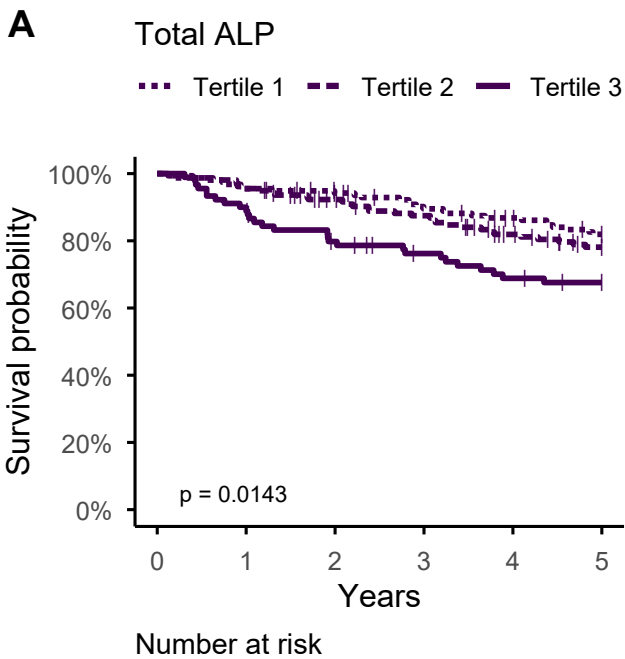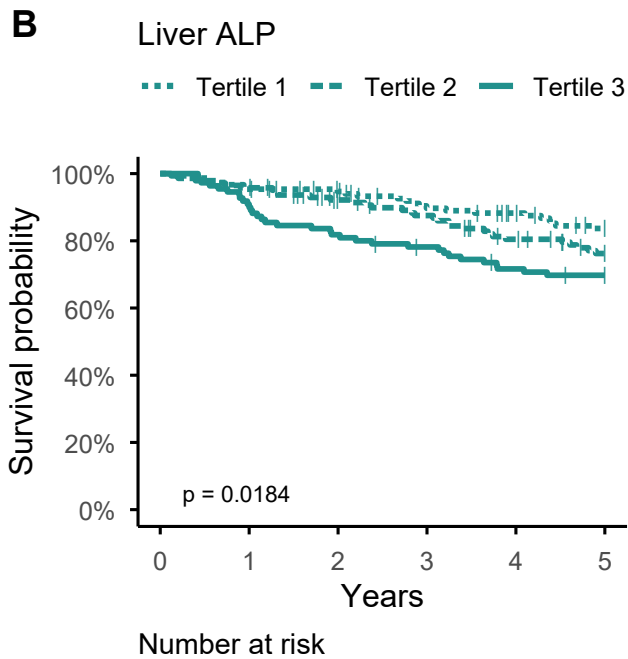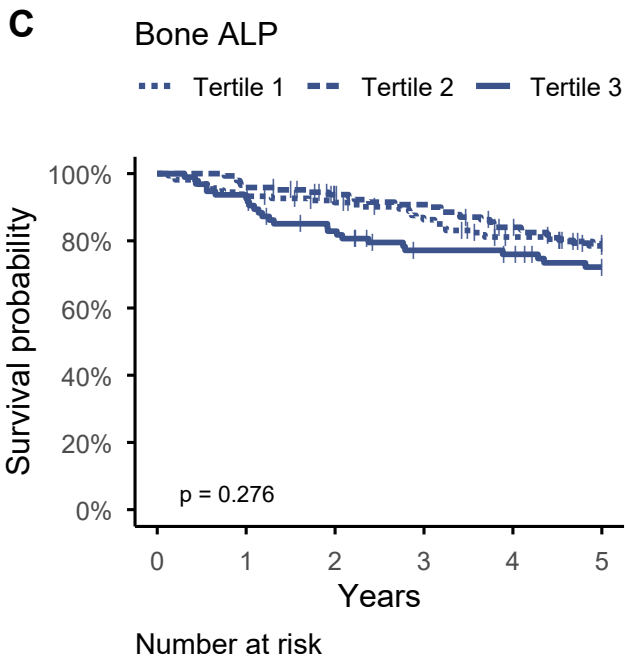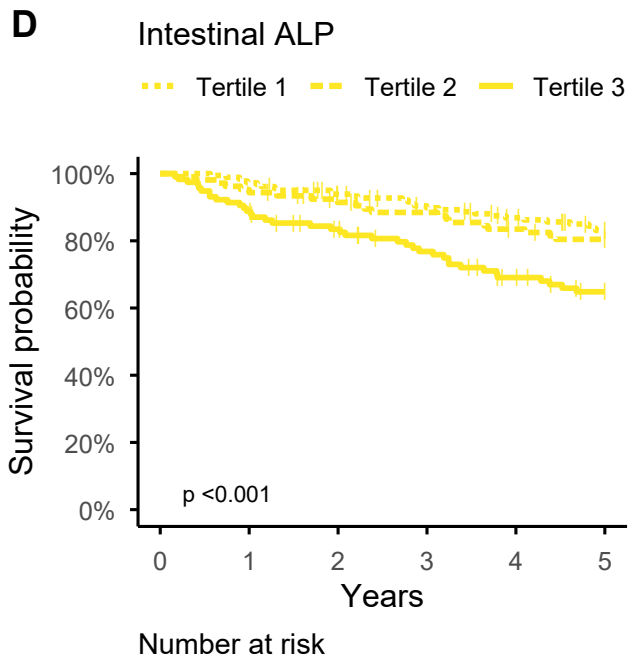

Supplement: sfag078_Supplemental_Files [file sfag078_supplemental_files.zip › Suppl_Fig_S2_KM_G1-5D.pdf]
